# Manganese Depletion Leads to Multisystem Changes in the Transcriptome of the Opportunistic Pathogen *Streptococcus sanguinis*

**DOI:** 10.3389/fmicb.2020.592615

**Published:** 2020-11-05

**Authors:** Tanya Puccio, Karina S. Kunka, Bin Zhu, Ping Xu, Todd Kitten

**Affiliations:** Philips Institute for Oral Health Research, Virginia Commonwealth University, Richmond, VA, United States

**Keywords:** *Streptococcus sanguinis*, infective endocarditis, manganese, transcriptome, fermentor, carbon catabolite repression, CcpA

## Abstract

*Streptococcus sanguinis* is a primary colonizer of teeth and is typically considered beneficial due to its antagonistic relationship with the cariogenic pathogen *Streptococcus mutans*. However, *S. sanguinis* can also act as an opportunistic pathogen should it enter the bloodstream and colonize a damaged heart valve, leading to infective endocarditis. Studies have implicated manganese acquisition as an important virulence determinant in streptococcal endocarditis. A knockout mutant lacking the primary manganese import system in *S. sanguinis*, SsaACB, is severely attenuated for virulence in an *in vivo* rabbit model. Manganese is a known cofactor for several important enzymes in *S. sanguinis*, including superoxide dismutase, SodA, and the aerobic ribonucleotide reductase, NrdEF. To determine the effect of manganese depletion on *S. sanguinis*, we performed transcriptomic analysis on a Δ*ssaACB* mutant grown in aerobic fermentor conditions after the addition of the metal chelator EDTA. Despite the broad specificity of EDTA, analysis of cellular metal content revealed a decrease in manganese, but not in other metals, that coincided with a drop in growth rate. Subsequent supplementation with manganese, but not iron, zinc, or magnesium, restored growth in the fermentor post-EDTA. Reduced activity of Mn-dependent SodA and NrdEF likely contributed to the decreased growth rate post-EDTA, but did not appear entirely responsible. With the exception of the Dps-like peroxide resistance gene, *dpr*, manganese depletion did not induce stress response systems. By comparing the transcriptome of Δ*ssaACB* cells pre- and post-EDTA, we determined that manganese deprivation led to altered expression of diverse systems. Manganese depletion also led to an apparent induction of carbon catabolite repression in a glucose-independent manner. The combined results suggest that manganese limitation produces effects in *S. sanguinis* that are diverse and complex, with no single protein or system appearing entirely responsible for the observed growth rate decrease. This study provides further evidence for the importance of this trace element in streptococcal biology. Future studies will focus on determining mechanisms for regulation, as the multitude of changes observed in this study indicate that multiple regulators may respond to manganese levels.

## Introduction

*Streptococcus sanguinis* is a facultative anaerobe that is typically found in much greater abundance at healthy oral sites than in carious lesions or diseased gingiva (Stingu et al., 2008; Belda-Ferre et al., 2012; Griffen et al., 2012; Gross et al., 2012; Giacaman et al., 2015). The *S. sanguinis* genome encodes a variety of adhesins (Xu et al., 2007; Bensing et al., 2018) that allow it to act as one of the primary colonizers of the salivary pellicle (Socransky et al., 1977; Kolenbrander et al., 2010). It has the capacity to produce (Garcia-Mendoza et al., 1993; Kreth et al., 2008) and survive in (Xu et al., 2014) high concentrations of hydrogen peroxide (H_2_O_2_), which allows it to compete against the dental caries pathogen *Streptococcus mutans* (Kreth et al., 2005). These traits, which have evolved to ensure survival in the highly diverse oral cavity, also make *S. sanguinis* an opportunistic pathogen (Das et al., 2009; Turner et al., 2009; Bensing et al., 2019). Dental procedures (Kinane et al., 2005; Forner et al., 2006; Lockhart et al., 2008), routine oral hygiene practices (Silver et al., 1977, 1979; Moreillon and Que, 2004), mastication (Sreenivasan et al., 2017), and poor oral hygiene (Kholy et al., 2015) can all damage the oral mucosa, allowing bacteria to enter the bloodstream. *S. sanguinis* and certain other bacterial species can bind to cardiac vegetations composed of platelets and fibrin that form on damaged heart valves and endocardium (Lee et al., 2001), leading to the disease infective endocarditis (IE) (Moreillon et al., 2002; Widmer et al., 2006). Recent studies estimate that IE affects more than 40,000 people each year in the United States and kills 12–40% (Bor et al., 2013; Cahill et al., 2017; Jamil et al., 2019) due to complications such as congestive heart failure and stroke (Bashore et al., 2006). In the United States, prevention depends upon antibiotic prophylaxis prior to dental procedures for at-risk patients (Wilson et al., 2007). The economic burden, potential for side effects, and questionable efficacy (Dayer and Thornhill, 2018; Thornhill et al., 2018; Quan et al., 2020) of this practice, as well as the increasing prevalence of antibiotic resistance (Dodds, 2017) are all pressing concerns.

Manganese (Mn) is an essential human micronutrient and has been linked to virulence in many human pathogens, including streptococci (Kehres and Maguire, 2003; Papp-Wallace and Maguire, 2006; Eijkelkamp et al., 2015). Mn has been shown to play an important role in oxidative stress tolerance through several mechanisms: (i) serving as a cofactor for enzymes that break down reactive oxygen species (ROS), (ii) substituting for iron (Fe) in enzymes when cells experience oxidative stress in order to prevent Fenton reaction-mediated damage, and (iii) reacting with superoxide radicals when complexed with small molecules such as bicarbonate (Waters, 2020).

Previous work from our lab established that the ABC transporter SsaACB is important for Mn transport and essential for virulence in a rabbit model of IE (Crump et al., 2014; Baker et al., 2019; Murgas et al., 2020). In *S. sanguinis*, Mn acts as a cofactor for superoxide dismutase (SodA) (Parker and Blake, 1988; Poyart et al., 1988) and the aerobic class 1b ribonucleotide reductase (NrdEF) (Makhlynets et al., 2014; Rhodes et al., 2014). Loss of SodA activity alone cannot account for the reduction in virulence (Crump et al., 2014). NrdEF activity is essential for virulence (Rhodes et al., 2014), but it is likely that these are not the only two Mn-cofactored enzymes or Mn-dependent pathways in *S. sanguinis*. In a previous microarray analysis of Mn depletion in the closely related species *Streptococcus pneumoniae* (Ogunniyi et al., 2010), it was found that only a few genes were differentially expressed in response to either deletion of the pneumococcal SsaB ortholog PsaA, or growth in media without supplemental Mn. However, these data alone are insufficient to explain the decreased growth of these mutants in low-Mn media. In this study, we sought to determine the overall effect of Mn depletion on the transcriptome of *S. sanguinis* in an attempt to identify other Mn-dependent pathways. Here we report that while there were some similarities with this previous study, we found a larger number of differentially expressed genes, providing new insights into the role of Mn in streptococci.

## Results

### Selection of Fermentor Growth Conditions for Mn Depletion

For this study, we were interested in measuring transcriptional changes resulting from Mn depletion in metabolically active cells. We also wanted to examine the cells as they transitioned from Mn replete conditions to Mn insufficiency, a task that would most easily have been accomplished by addition of a strong and selective Mn chelator to growing cells. However, we were aware of no such chelator. We therefore explored the use of the non-specific chelator EDTA in conjunction with an Δ*ssaACB* mutant. This mutant was previously found to be deficient in Mn and Fe transport and aerobic growth in low-Mn media (Murgas et al., 2020).

We achieved reproducible, large-scale growth in a fermentor using Brain Heart Infusion (BHI) broth. Typical chemostat conditions (Burne and Chen, 1998) could not be identified that supported growth of the SK36 wild-type (WT) strain but not the Δ*ssaACB* mutant, even when aeration was increased (data not shown). However, we found that when the dilution rate was increased to 0.875 vessel volumes per h, addition of 100 μM EDTA to both the fermentor vessel and media carboy dramatically reduced the optical density (OD_840__–__910_) of the Δ*ssaACB* mutant cultures (Supplementary Figure S1A), while not affecting the WT strain (Supplementary Figure S1B). The effect of EDTA addition on the OD of the Δ*ssaACB* cultures typically became apparent after 38 min (Supplementary Figure S1 inset). The addition of EDTA slowed the growth of Δ*ssaACB* but did not kill the cells entirely because when the media pumps were shut off ∼80 min post-EDTA addition, the OD began to increase immediately (data not shown).

**FIGURE 1:**
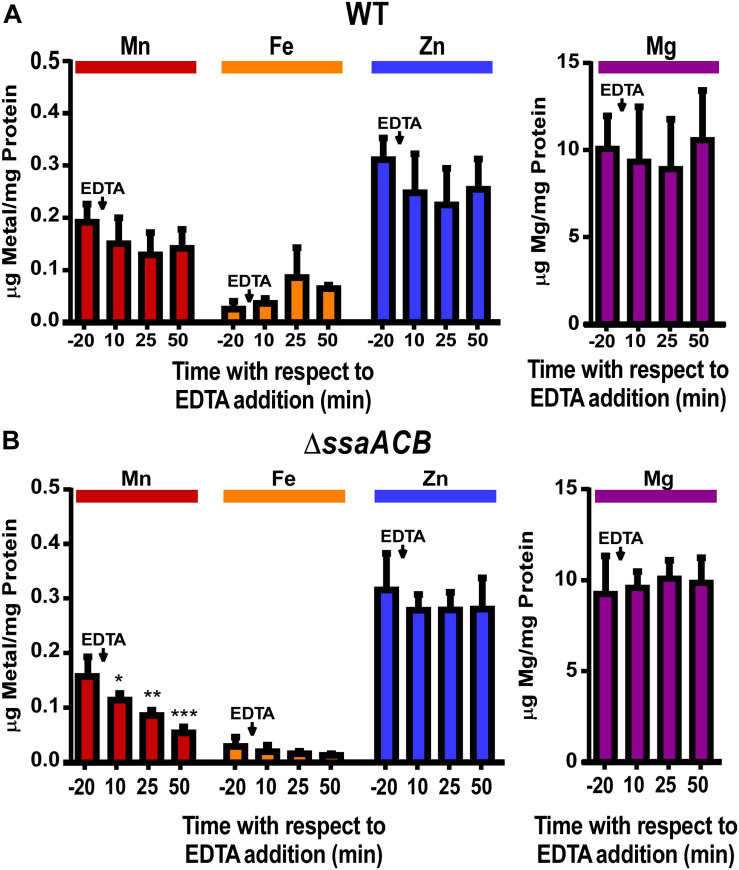
Effect of EDTA on metal content of fermentor-grown WT and Δ*ssaACB* cells. Aerobic fermentor-grown WT **(A)** and Δ*ssaACB* **(B)** cells were collected at each time point and analyzed for cellular metal content using ICP-OES. Means and standard deviations of three replicates are shown. Significance was determined either by repeated measures ANOVA or by one-way ANOVA if matching was not effective, with a Tukey-Kramer multiple comparisons test to T_–__20_. *P ≤ 0.05, **P ≤ 0.01, ***P ≤ 0.001. Time points not labeled were not significantly different from T_–__20_. For Fe, two T_–__20_ replicates in **(A)** and at least two replicates for each time point in **(B)** were below the limit of detection.

To determine if a lack of available Mn caused the EDTA-dependent reduction in the Δ*ssaACB* growth rate, samples of both WT and Δ*ssaACB* were collected at T_–__20_, T_10_, T_25_, and T_50_, where 100 μM EDTA was added to the vessel at T_0_ (Supplementary Figure S1). Washed cells were analyzed using inductively coupled plasma optical emission spectroscopy (ICP-OES) (Figure 1). EDTA addition to WT did not significantly alter cellular levels of any of the four metals measured—Mn, Fe, zinc (Zn), or magnesium (Mg) (Figure 1A). Mn was the only metal significantly reduced in the post-EDTA samples as compared to pre-EDTA for Δ*ssaACB* (Figure 1B). Fe levels were low in the Δ*ssaACB* mutant (Figure 1B), consistent with previous results that this mutant transports less Fe than WT (Murgas et al., 2020). Fe, Zn, and Mg levels were not significantly affected by EDTA addition (Figure 1). Cobalt and copper levels were at or below the limit of detection in both strains (data not shown).

As another test of metal specificity, 100 μM of either Mn^2+^ or Fe^2+^ was added to the vessel 70 mins post-EDTA addition. The addition of Mn^2+^ eliminated, and then reversed, the post-EDTA decline in OD, while Fe^2+^ had no discernible effect (Supplementary Figure S2). The metal content of samples collected 10 mins after addition of Mn^2+^ or Fe^2+^ (T_80_) revealed that both Mn and Fe were taken up by cells, resulting in significantly higher levels than at T_–__20_ (Supplementary Figure S3). Although neither Zn nor Mg levels were significantly affected by addition of EDTA (Figure 1), 100 μM of either Zn^2+^ or Mg^2+^ was added at T_70_ for at least two fermentor runs each and, like Fe^2+^, neither produced any apparent effect (data not shown).

### Overview of Transcriptional Response of *S. sanguinis* to Mn Depletion

**FIGURE 2:**
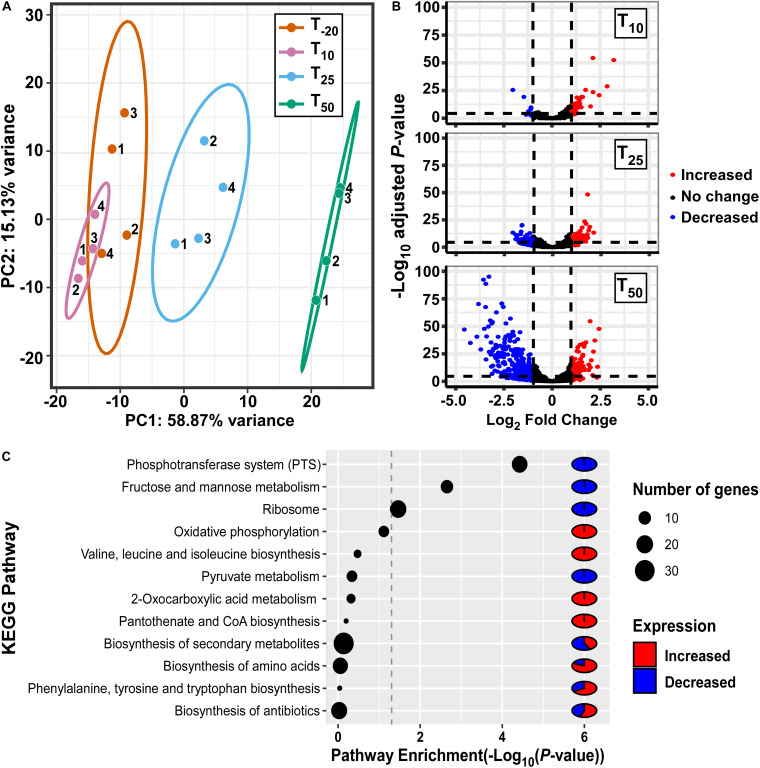
Analysis of gene expression in fermentor-grown *S. sanguinis* Δ*ssaACB* mutant cells. **(A)** Principal component analysis of the RNA-seq samples as determined by the pcaExplorer package for R. Replicates are labeled by fermentor run number. Ellipses are drawn around the 95% confidence interval for each time point. **(B)** Volcano plots comparing each post-EDTA time point to T_–__20_ were generated using log_2_ fold changes from Geneious in the EnhancedVolcano package for R. Genes that are upregulated in the post-EDTA time point are positive on the x-axis (right) and those that are downregulated are negative (left). Genes exhibiting |log_2_ fold change| >1 are depicted by either red (>1) or blue (<1) spheres. **(C)** Pathway enrichment analysis of differentially expressed genes at T_50_ using DAVID classification of genes and KEGG annotations.

In order to assess the impact of Mn depletion on the *S. sanguinis* transcriptome, RNA sequencing (RNA-seq) analysis was performed on Δ*ssaACB* fermentor samples collected at the same time points as above (Supplementary Figure S1A). Principal component analysis (PCA) revealed that the samples from each time point clustered together, indicating minimal variation between independent replicates (Figure 2A). The T_10_ samples overlapped slightly with T_–__20_, indicating few early changes in gene expression. The dissimilarities of the RNA-seq profiles were greater at T_25_ and T_50_, suggesting that EDTA treatment increasingly affected the gene expression of Δ*ssaACB* during the period tested.

Volcano plot analysis of differentially expressed genes (DEGs; defined as | log_2_| ≥ 1, adjusted *P*-value ≤ 0.05) comparing post-EDTA time points to the pre-EDTA time point revealed that there were only 48 (2.1%) and 139 (6.1%) DEGs at T_10_ and T_25_, respectively (Figure 2B). In contrast, at 50 mins post-EDTA, 407 genes (17.9%) were differentially expressed, with a number of genes more severely downregulated (Figure 2B). Consistent with these results, the growth rate of Δ*ssaACB* decreased dramatically between T_25_ and T_50_ (Supplementary Figure S1A).

Gene classification analysis of DEGs at T_50_ using KEGG annotations revealed that genes involved in sugar metabolism were highly enriched, with phosphotransferase systems (PTS), fructose/mannose transport, and pyruvate metabolism all in the top 12 most highly enriched pathways (Figure 2C). Other highly enriched pathways included amino acid metabolism, ribosomes, oxidative phosphorylation (ATP synthases), and biosynthesis of coenzymes and various secondary metabolites (Figure 2C).

RNA-seq trends for several genes of interest with moderate to high expression level changes were validated by measuring mRNA levels of fermentor samples via quantitative reverse transcriptase polymerase chain reaction (qRT-PCR) (Supplementary Figure S4). The relative expression levels observed in the qRT-PCR experiments largely replicated the trends observed in the RNA-seq analysis.

In the following sections, we highlight results we believe to be most important.

### Regulation of Metal Transport Genes

**FIGURE 3:**
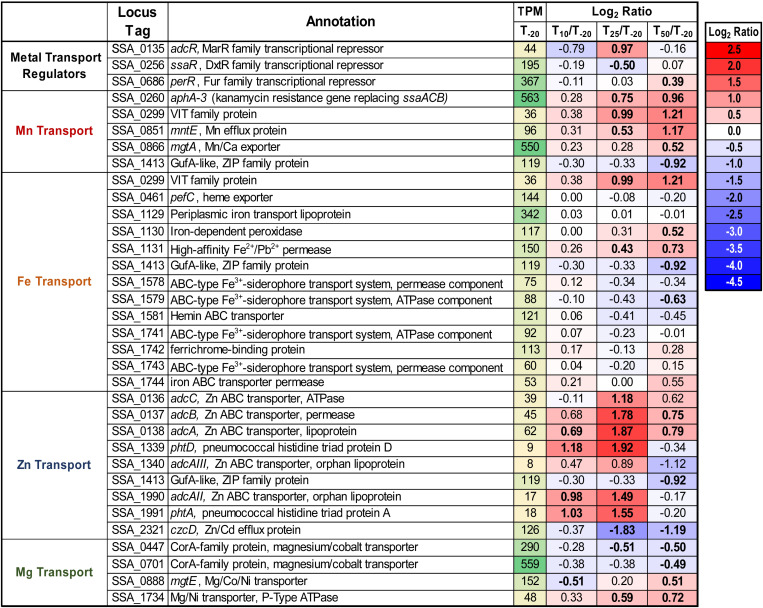
Expression of metal transport genes post-Mn depletion. Putative metal transport genes are depicted with their average transcripts per million reads (TPM) at T_–__20_ and log_2_ fold change values for each post-EDTA time point. TPM values greater than 1,000 are full saturation (green). Positive log_2_ fold change values (red) indicate genes upregulated after Mn depletion as compared to T_–__20_, while negative values (blue) indicate downregulated genes. Values in bold indicate significant changes in expression by adjusted *P*-value (≤0.05).

As seen in Figure 1, Mn was the only tested metal whose cellular concentration was decreased upon addition of EDTA to Δ*ssaACB* cells. To further investigate the impact of EDTA on metal transport, we examined the expression of metal transport genes (Figure 3). The kanamycin (Kan) resistance gene *aphA*-3 that replaced the Mn transporter operon, *ssaACB*, in this mutant strain was upregulated in all three post-EDTA time points (Figure 3). This is consistent with previous results from our lab showing Mn-dependent repression of SsaB expression as measured by western blot (Crump et al., 2014).

Given that the cells were Mn-depleted after EDTA addition (Figure 1), it was surprising to see the upregulation at T_50_ of genes encoding putative orthologs of Mn-export proteins MntE (Rosch et al., 2009; Martin and Giedroc, 2016) and MgtA (Martin et al., 2019) of the closely related species *S. pneumoniae* (Figure 3). Expression of *mntE* was found to be constitutive (Martin and Giedroc, 2016) and *mgtA* expression was found to be positively regulated by Mn through a metal-dependent riboswitch (Martin et al., 2019). We therefore sought to test whether Δ*mntE* and Δ*mgtA* mutants generated previously in *S. sanguinis* (Xu et al., 2011) exhibited increased Mn sensitivity relative to WT as expected based on previous findings in *S. pneumoniae* (Rosch et al., 2009; Martin et al., 2019). The Δ*mgtA* mutant grew as expected, with a lower final density than WT in BHI with 2 mM added Mn (data not shown). The Δ*mntE* mutant, however, grew just like WT, which was unlike the findings in *S. pneumoniae* (data not shown). We tested up to 10 mM Mn in BHI and Todd Hewitt broth with 1% yeast extract and could not find a Mn concentration that prevented growth of either the Δ*mntE* mutant or WT (data not shown). Initial metal analysis revealed that the Δ*mntE* mutant accumulated slightly more Mn than WT (data not shown). These results indicate that *S. sanguinis* may primarily use MgtA to export excess Mn, and MntE may function differently in *S. sanguinis* than in *S. pneumoniae*. Future studies will elucidate the function of these putative exporters and their transcriptional regulation in *S. sanguinis*.

As seen in Figure 1, cellular Zn levels in Δ*ssaACB* were not significantly altered by EDTA addition, despite the high affinity of this chelator for Zn (Perrin and Dempsey, 1974). Zn level maintenance may be due to the higher levels of Zn than Mn in BHI (1.7 ± 0.02 vs. 0.02 ± 0.003 μg mL^–1^, respectively) (Murgas et al., 2020) or the regulation of Zn transporter genes. *S. sanguinis* possesses orthologs of the Zn ABC transporter AdcCBA of *S. pneumoniae* (Dintilhac and Claverys, 1997), and all three genes were upregulated post-EDTA (Figure 3). Expression of the gene encoding the Zn^2+^ and Cd^2+^ efflux protein, CzcD (Nies, 1992), decreased after EDTA addition (Figure 3). Thus, cellular Zn levels appear to have been maintained during EDTA treatment by decreasing export of intracellular Zn and increasing import of any remaining bioavailable Zn through regulation of Zn transporters.

We also examined the regulation of other putative Zn-transport proteins. In *S. pneumoniae*, AdcAII and several histidine triad proteins also contribute to Zn transport. AdcAII is an orphan lipoprotein of the AdcCBA system (Bayle et al., 2011; Plumptre et al., 2014) and PhtD is a histidine triad protein encoded adjacent to AdcAII (Bersch et al., 2013; Kallio et al., 2014). *S. sanguinis* has two genes, SSA_1340 and SSA_1990, that encode proteins similar to AdcAII, and each is also adjacent to putative histidine triad protein genes, SSA_1339 or SSA_1991. Because AdcAII is more similar to SSA_1990, we have named this protein AdcAII, whereas we have designated SSA_1340 as AdcAIII. Consistent with a potential role in Zn uptake, all four of these genes were upregulated at T_25_ (Figure 3). The relative contribution of each of these proteins to Zn import remains to be determined, although we hypothesize that the upregulation of these genes contributes to the tight maintenance of Zn levels in cells post-EDTA.

Expression data for additional putative metal transporters may be found in Figure 3.

### Examination of Known Mn-Cofactored Enzymes

#### Superoxide Dismutase

**FIGURE 4:**
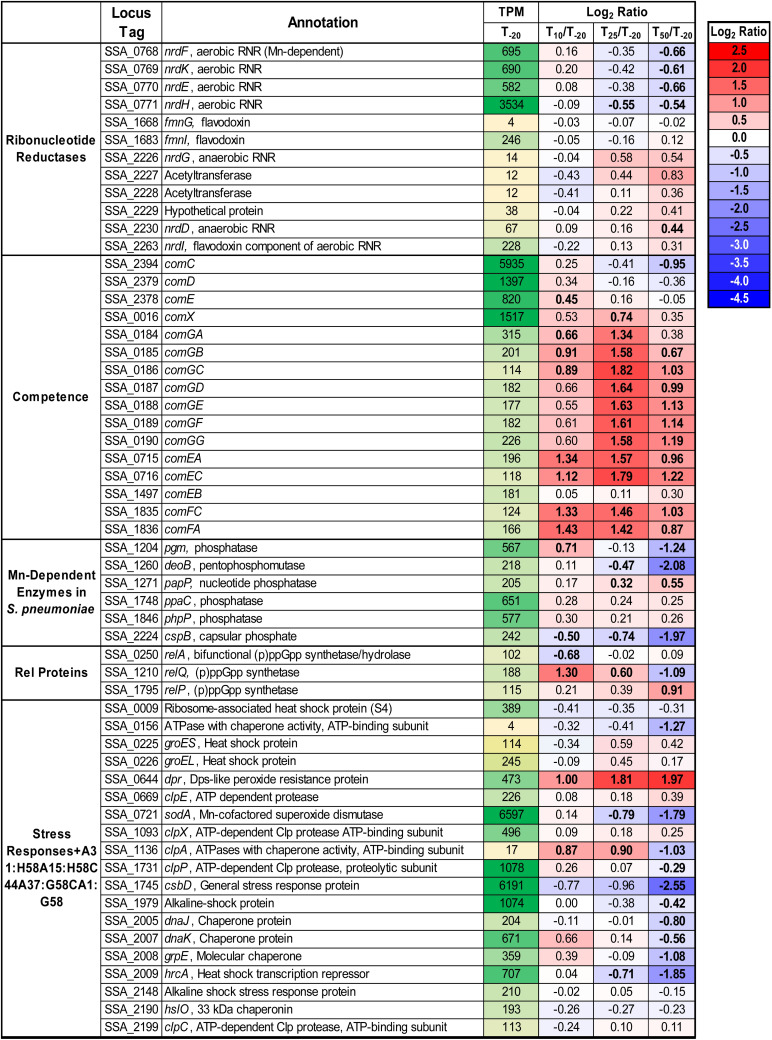
Expression of select genes post-Mn depletion. Selected genes of interest are depicted with their average TPM at T_–__20_ and log_2_ fold change values for each post-EDTA time point. TPM values greater than 1,000 are full saturation (green). Positive log_2_ fold change values (red) indicate genes upregulated after Mn depletion as compared to T_–__20_, while negative values (blue) indicate downregulated genes. Values in bold indicate significant changes in expression by adjusted *P*-value (≤0.05).

*S. sanguinis* possesses a single superoxide dismutase, SodA (Xu et al., 2007), and it is Mn-cofactored (Crump et al., 2014). Our previous study indicated that reduced SodA activity could account for only a portion of the reduced virulence and aerobic serum growth of the Δ*ssaB* mutant (Crump et al., 2014). Expression of *sodA* decreased significantly at both T_25_ and T_50_ despite constant air input (Figure 4), which may be due to Mn-dependent positive regulation of transcription (Jakubovics et al., 2002; Eijkelkamp et al., 2014). Given that the fermentor growth conditions do not exactly replicate our previous *in vitro* or *in vivo* assays, we wondered whether SodA would be important here. To answer this question, we grew our Δ*sodA* knockout mutant in the fermentor under the same conditions without EDTA. The Δ*sodA* mutant grew similarly to WT (Supplementary Figure S5), indicating that Mn-dependent SodA activity is not essential for aerobic growth under these conditions. While this does not rule out the possibility that reduced SodA activity post-Mn depletion contributed to the reduced growth rate of Δ*ssaACB*, it established that it was not the sole cause, thus encouraging us to investigate other possibilities.

#### Class Ib Aerobic Ribonucleotide Reductase

The other known Mn-cofactored enzyme in *S. sanguinis* is the aerobic class Ib ribonucleotide reductase (RNR), NrdEF (Makhlynets et al., 2014; Rhodes et al., 2014). RNR enzymes catalyze the production of deoxynucleotides from the corresponding ribonucleotides. It was previously found that mutant strains lacking this enzyme were unable to grow in aerobic conditions, whether in serum or BHI. These studies also suggested that Fe could not substitute for Mn as an RNR cofactor *in vivo*, despite its ability to do so *in vitro*. In addition to a likely decrease in activity, expression of *nrdHEKF* was downregulated after Mn depletion (Figure 4). We recently analyzed the metabolome of *S. sanguinis* cells under the same conditions as this study (Puccio et al., 2020). Levels of detected deoxynucleosides and deoxynucleotides in cells increased or remained constant after Mn depletion. Thus, while NrdEF requires Mn for activity, our data suggest that deoxynucleotides may not be a limiting factor for growth in our study.

#### Mn-Dependent Phosphatases

In the related species *S. pneumoniae*, there are six additional enzymes (Figure 4) that have been found to have Mn cofactors (Kuipers et al., 2016; Martin et al., 2017). Orthologs of all six enzymes are encoded in the *S. sanguinis* genome. Although each is a reciprocal best hit with its *S. pneumoniae* counterpart by NCBI BlastP, their functions have not been confirmed. Pgm and CspB are phosphatases that have been implicated in capsule biosynthesis in *S. pneumoniae*, although *S. sanguinis* lacks a true capsule. DeoB is a phosphopentomutase that connects the pentose phosphate pathway to purine biosynthesis and was also significantly downregulated at T_50_. Expression of *papP*, encoding a nucleotide phosphatase, was significantly increased at the later time points and has been shown to affect membrane lipid homeostasis (Kuipers et al., 2016). A significant morphological difference was observed in Δ*papP* mutants in *S. pneumoniae*, but Δ*ssaACB* cells from the T_50_ sample did not appear morphologically different from cells at T_–__20_ (data not shown). Of note, we observed changes in fatty acid synthesis under these same fermentor growth conditions (Puccio et al., 2020), suggesting that activity of PapP may be reduced but not to the extent that it substantially affects morphology.

Genes encoding the other two phosphatases, PhpP and PpaC, were not differentially expressed at any time point (Figure 4). While this indicates the lack of a Mn-dependent regulation mechanism, it does not rule out the possibility that their activity was decreased due to reduction of Mn. PhpP is a serine/threonine protein phosphatase that is a key regulator of cell division and has been shown to be regulated by the bioavailable Zn:Mn ratio in *S. pneumoniae* (Martin et al., 2017). While the Zn:Mn ratio did increase over time in our study (Table 1), morphology did not differ from T_–__20_ (data not shown), indicating that PhpP may not be affected by Mn limitation under these conditions. In our recent study, loss of PhpP did not significantly affect the growth of *S. sanguinis* in serum, a Mn-limited medium (Zhu et al., in press), which indicates that it is likely not responsible for the growth rate decrease observed here. The last phosphatase, PpaC, is essential for *S. sanguinis* (Xu et al., 2011), so if PpaC activity was decreased due to Mn depletion, this could have contributed to the decreased growth rate phenotype observed post-EDTA. Further studies utilizing the knockout mutants of each non-essential phosphatase (Xu et al., 2011) or an approach such as CRISPR interference (Shields et al., 2020) for PpaC would enhance our understanding of the relative contributions of each phosphatase to the growth and morphology of *S. sanguinis*.

**TABLE 1:** Zn:Mn ratios in fermentor grown cells.

Strain	T_–__20_	T_10_	T_25_	T_50_
WT	1.64	1.66	1.75	1.81
Δ*ssaACB*	2.02	2.47*	3.30*	5.36*

*Values indicate ratios of Zn concentrations as compared to those of Mn. Concentrations were determined by ICP-OES and significance was determined by one-way ANOVA with a Tukey-Kramer multiple comparisons test vs. T*_–_*_20_ where* * *indicates P ≤ 0.05.*

#### (p)ppGpp Hydrolase Domain

In streptococci and enterococci, Mn acts as a cofactor for the hydrolase domain of the bifunctional (p)ppGpp synthetase/hydrolase, RelA (also called RSH for RelA/SpoT Homologs) (Mechold et al., 1996). As an alarmone, (p)ppGpp serves as an effector of the stringent response in bacteria (Irving and Corrigan, 2018). Expression of *relA* was unchanged after EDTA addition, and expression of the other two small alarmone synthetase genes, *relP* and *relQ* (Lemos et al., 2007), were significantly increased and decreased, respectively (Figure 4). Both RelP and RelQ were found to produce less (p)ppGpp than RelA in *S. mutans* (Lemos et al., 2007) and appear to be important during different environmental conditions or growth stages in gram-positive bacteria (Yang et al., 2019).

**FIGURE 5:**
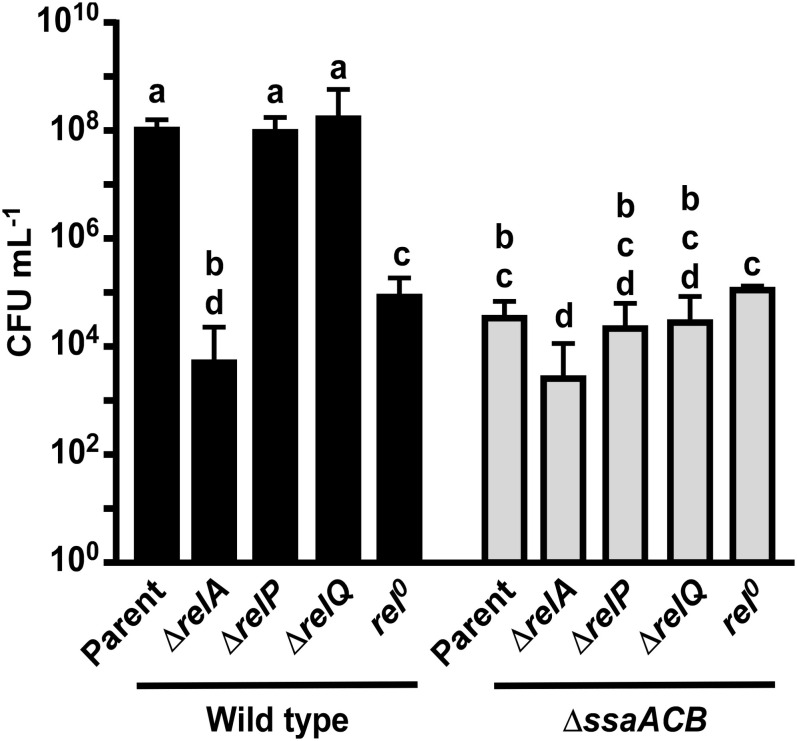
Aerobic serum growth of *rel* mutants. Various *rel* mutants were grown for 24 h in 6% O_2_ in pooled rabbit serum. The means and standard deviations of at least three replicates are displayed. Significance was determined by one-way ANOVA with a Tukey-Kramer multiple comparisons test. T_24_ bars with the same letter are not significantly different from each other. Initial culture concentrations were measured (not shown) and found to be not significantly different by one-way ANOVA.

In an attempt to determine whether loss of hydrolase activity in RelA could account for the phenotypes we observed in Mn-depleted cells, we attempted to construct a hydrolase-deficient mutant by altering specific residues (R44, H62, T151) shown by Hogg et al. (2004) to be important for (p)ppGpp hydrolase, but not synthetase activity. Similar to Kaspar et al. (2016), we were unable to generate any of the three point mutants without unintended mutations arising in other regions of the gene (data not shown). This indicates that hydrolase activity may be essential for growth of *S. sanguinis*. We then obtained strains from the comprehensive *S. sanguinis* mutant knockout library created by Xu et al. (2011) that were deleted for each of the *rel* genes. We also generated a *rel*^0^ strain by knocking out all three *rel* genes utilizing a markerless mutagenesis system (Xie et al., 2011; Cheng et al., 2018). We also made these *rel* knockout mutants in the Δ*ssaACB* background. We then assessed the growth of these mutants in aerobic serum-our *in vitro* model for infective endocarditis (Crump et al., 2014). As shown in Figure 5, neither Δ*relP* nor Δ*relQ* grew to a density that differed significantly from its parent strain, whether in the WT or Δ*ssaACB* background. Likewise, in both backgrounds Δ*relA* was more severely attenuated than *rel*^0^, suggesting that is it more detrimental to lose activity of RelA than to lack all (p)ppGpp.

### Assessment of Stress and Stress Responses in Mn-Depleted Cells Through Gene Expression

We next sought to determine whether the RNA-seq data suggested anything concerning stresses experienced by the cells. Expression of various stress response genes were assessed, and most were either downregulated or unchanged at T_50_ (Figure 4), indicating that the reduced growth rate is likely not due to an overwhelming stress response. The only stress response-related gene to show a significant increase in expression at T_50_ was that encoding the Dps-like peroxide resistance protein, Dpr (Yamamoto et al., 2000). Dpr is a ferritin-like protein that has been shown to be imperative for oxidative stress tolerance in several streptococci (Yamamoto et al., 2000, 2002; Pulliainen et al., 2003; Brenot et al., 2005), including *S. sanguinis* SK36 (Xu et al., 2014).

**FIGURE 6:**
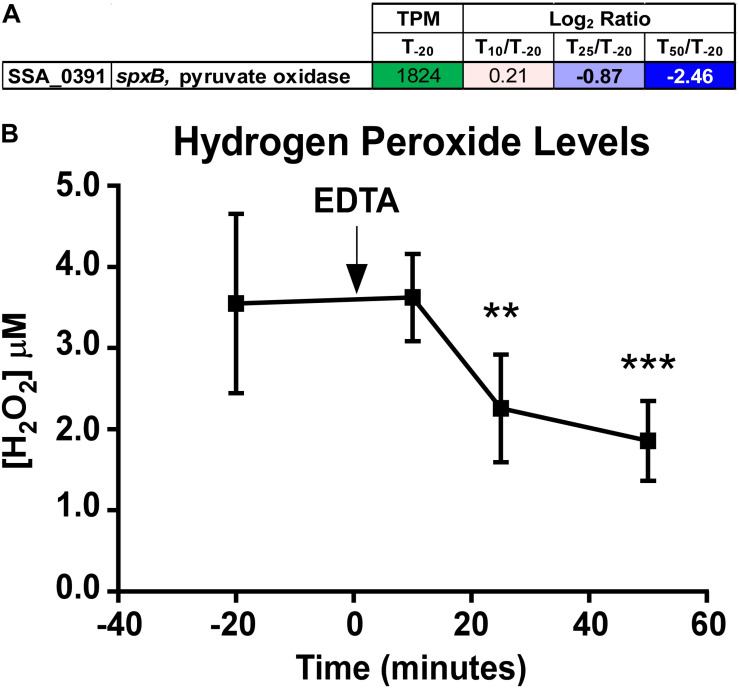
Quantitation of *spxB* expression and hydrogen peroxide in Δ*ssaACB* fermentor cultures. **(A)** Expression of the *spxB* gene in fermentor-grown Δ*ssaACB* cells as determined by RNA-seq analysis. Average TPM at T_–__20_ and log_2_ fold change values for each after Mn depletion time point are displayed. TPM values greater than 1,000 are full saturation (green). Positive log_2_ fold change values (red) are genes upregulated in after Mn depletion samples as compared to T_–__20_, while negative values (blue) indicate downregulated genes. Values in bold indicate significant changes in expression by adjusted *P*-value (≤0.05). **(B)** H_2_O_2_ levels of the BHI culture supernatant were measured at each time point. Means and standard deviations of at least four replicates are shown. Significance was determined by one-way ANOVA with a Tukey-Kramer multiple comparisons test, comparing each post-EDTA time point to T_–__20_. **P ≤ 0.01, ***P ≤ 0.001.

*S. sanguinis* is known to generate copious amounts of H_2_O_2_, presumably to more effectively compete against other oral species, such as the caries-forming pathogen *S. mutans* (Kreth et al., 2005; Chen et al., 2011). Simple Mn compounds have been reported to prevent oxidative stress by catalyzing the decomposition of H_2_O_2_ (Liochev and Fridovich, 2004) and superoxide (Barnese et al., 2008, 2012). We observed a significant decrease in expression of the gene encoding the H_2_O_2_-generating enzyme pyruvate oxidase, *spxB* (Spellerberg et al., 1996; Chen et al., 2011), at T_25_ and T_50_ (Figure 6A). To determine whether the decreased growth rate of the Δ*ssaACB* strain during aerobic fermentor growth after Mn depletion was due to excess H_2_O_2_ generation or the inability of cells to cope with H_2_O_2_ without Mn, H_2_O_2_ levels were measured in spent supernatant. Concentrations ranged between 1 and 5 μM (Figure 6B), far lower than has been observed in previous studies employing SK36 (Kreth et al., 2008) despite the constant influx of air into the vessel. H_2_O_2_ levels also decreased significantly at T_25_ and T_50_ as compared to T_–__20_ (Figure 6B), which correlates with the decreased expression of *spxB* (Figure 6A). These results indicate that oxidative stress related to excess H_2_O_2_ levels is unlikely to be the cause of the growth rate decrease observed upon Mn depletion.

### Analysis of Carbon Catabolite Repression and Sugar Transport

Examination of DEG clusters revealed that the majority of those thought to transport sugars were downregulated (Supplementary Table S2), and of these, the majority belonged to the PTS family, which is regulated by carbon catabolite repression (CCR). CCR is a regulatory mechanism that gives bacteria the ability to utilize carbon sources in order of preference (Gorke and Stulke, 2008). In gram-positive bacteria, a carbon catabolite protein such as CcpA binds to catabolite responsive elements (*cre*) and represses transcription of genes encoding non-preferred carbon source transport and utilization systems (Warner and Lolkema, 2003). To determine the extent to which CcpA binding could be responsible for the observed downregulation, *cre* sites identified previously by RegPrecise (Novichkov et al., 2013) and by our custom searches were collected and compared. Using these methods, 393 putative binding sites were identified (Supplementary Table S2). Several PTS and sugar ABC transport genes were predicted to have 5’ *cre* sites, the majority of which were downregulated at T_50_. Other genes known to be CcpA-regulated in SK36, such as *spxB* (Zheng et al., 2011; Redanz et al., 2018), were downregulated as well. This is surprising given that the glucose-containing media was replenished at a constant and rapid rate throughout the experiment, indicating that there could be a Mn-related mechanism for CcpA repression. Cells also did not appear to be starved for glucose; when excess glucose (1.8%) was added to the media, the post-EDTA growth rate was similar to that of normal BHI, which contains 0.2% glucose (data not shown). This is not entirely unexpected, as it has been established by Redanz et al. (2018) that CcpA repression of *spxB* in *S. sanguinis* is glucose-independent.

Several CcpA-regulated genes were annotated as amino acid transporters and synthetases, which led us to examine the expression of genes with these putative functions. Many of these genes were differentially expressed (Supplementary Figure S6), which may be due to the intertwined nature of amino acid and carbohydrate metabolism (Radin et al., 2016). We also observed decreased expression of large, contiguous loci encoding ethanolamine utilization (Fox et al., 2009; Kaval and Garsin, 2018; Kaval et al., 2019), a type IV pilus system (Chen et al., 2019), and CRISPR-associated proteins (Makarova et al., 2015; Gong et al., 2020; Supplementary Figure S7), which are all also tied to CcpA-regulation (Bai et al., 2019).

## Discussion

Only two enzymes have been confirmed to be Mn-dependent in *S. sanguinis*, and few others have been identified in other streptococci. Despite this, we observed changes in a wide variety of systems after Mn depletion of the Δ*ssaACB* mutant using EDTA. One possible explanation for this discrepancy is that Mn binds with low affinity to most proteins, resulting in Mn loss or replacement during purification. In fact, initial studies of the aerobic class Ib RNR identified Fe as the exclusive cofactor based on RNR activity *in vitro* and the fact that Fe was present in many different bacterial RNRs heterologously expressed in *E. coli*. Only later was it discovered that these enzymes were Mn-cofactored when natively expressed (Cotruvo and Stubbe, 2012), and despite the *in vitro* activity of both forms of the *S. sanguinis* RNR, only the Mn-cofactored version was active *in vivo* (Makhlynets et al., 2014; Rhodes et al., 2014). Additional Mn-dependent enzymes may have similarly escaped detection. Another possible explanation is that Mn depletion impacts several key regulatory systems, such as CCR and (p)ppGpp, which leads to changes in the expression of many different genes. Mn levels have been found to be related to each of these systems in other gram-positive bacteria (Kehres and Maguire, 2003; Colomer-Winter et al., 2017). Mn depletion does not appear to induce a traditional stress response, although expression of an oxidative stress tolerance protein, Dpr, significantly increased, which is consistent with a recent study on Mn depletion in *S. mutans* (Kajfasz et al., 2020). Here we highlight Mn-related systems we identified in this study of *S. sanguinis* for future investigation.

### Mn Depletion Leads to Glucose-Independent Changes in the Regulon of CcpA

Given that BHI contains glucose, it was expected that *S. sanguinis* would preferentially transport and utilize it as a preferred carbon source under standard fermentor conditions. This is supported by the fact that glucose levels decreased in the media after cell growth (Puccio et al., 2020) as well as by the high expression of putative glucose transporters SSA_1752, SSA_1918-1920, and SSA_1298-1300 (Ajdic and Pham, 2007) at T_–__20_ (Supplementary Table S2). Surprisingly, expression of nearly all sugar transport systems decreased after Mn depletion (Supplementary Table S2), despite constant levels of glucose in the cells (Puccio et al., 2020). CcpA is known to repress its own expression in a glucose-dependent manner (Bai et al., 2019), and yet much like the glucose transporters, *ccpA* expression was high at T_–__20_ and significantly decreased by T_50_ (Supplementary Table S2). Potential explanations could include: (i) 2 g/L glucose in BHI is not sufficient to induce CcpA repression; (ii) other regulatory mechanisms are preventing proper CCR under these conditions; or (iii) much like *spxB*, many other systems in *S. sanguinis* are subject to glucose-independent CcpA repression. Redanz et al. (2018) used 0.3% as the low-glucose condition in their study of CcpA repression of *spxB* in *S. sanguinis*, whereas Bai et al. (2019) used BHI alone (0.2% glucose) to observe differences in the transcriptome between the WT and Δ*ccpA S. sanguinis* strains. Thus, the glucose concentration in BHI may indeed be low, yet sufficient to induce some repression of its regulon.

It is interesting that Mn depletion leads to an apparent increase in CcpA repression because we are not aware of this having ever been reported. The strongest evidence for CcpA-dependent regulation is *spxB.* In *S. sanguinis*, *spxB* expression has been shown to be positively regulated by SpxA1 (Chen et al., 2012) and VicK (Moraes et al., 2014) and negatively regulated by CcpA (Zheng et al., 2011). The *spxA1* gene was in the top 10% of all genes based on expression at T_–__20_ and remained unchanged after EDTA addition (Supplementary Table S1), indicating that repression by CcpA is likely responsible for the decrease in *spxB* expression as opposed to changes in induction by SpxA1. The mechanism by which CcpA represses *spxB* expression in *S. sanguinis* is unique from other streptococci in that it is independent of glucose (Redanz et al., 2018). It was previously determined that Mn may play a role in *spxB* expression in *S. pneumoniae*, as a Δ*mntE* mutant in *S. pneumoniae* accumulated Mn and produced more H_2_O_2_ than WT under excess Mn conditions (Rosch et al., 2009).

**FIGURE 7:**
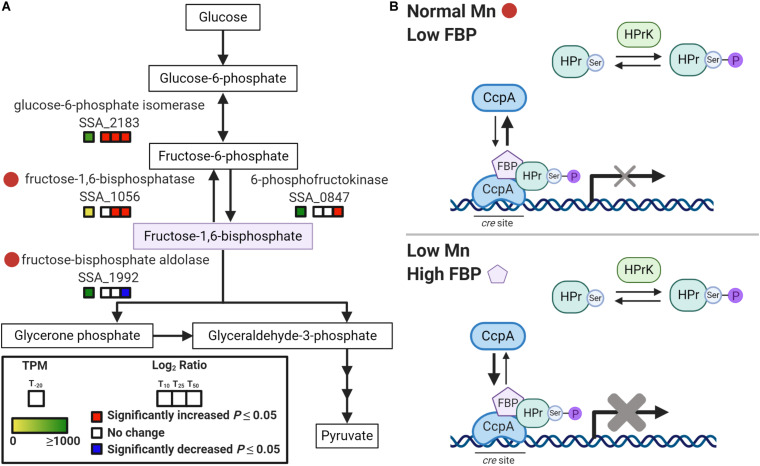
Model of Mn-dependent CcpA repression. **(A)** Depiction of a segment of the glycolysis and gluconeogenesis pathway in *S. sanguinis* from KEGG (Kanehisa and Goto, 2000). Red circles indicate potentially Mn-cofactored enzymes. Gene expression for each post-EDTA time point compared to T_–__20_ is indicated by the colored boxes. Significant changes (*P* < 0.05) are indicated by red (increased) or blue (decreased). TPM values at T_–__20_ are colored as indicated, with values ≥ 1,000 fully saturated (green). **(B)** Model of Mn-dependent CcpA repression based on CCR in Firmicutes as described by Gorke and Stulke (2008). In the top panel, normal Mn levels in BHI result in low FBP, which leads to less phosphorylation of HPr to HPr(Ser-P) by HPr kinase/phosphorylase (HPrK). With low FBP and HPr(Ser-P), CcpA exists mainly in its free state, unbound to *cre* sites in the DNA. This results in little to no repression of the CcpA regulon. The bottom panel depicts Mn depletion, where reduced activity of fructose-1,6-bisphosphatase and fructose-bisphosphate aldolase leads to an accumulation of FBP. This induces the phosphorylation of HPr so that it is primarily in the HPr(Ser-P) state. Increased FBP levels also enhance binding of CcpA to HPr(Ser-P) and to DNA. This results in augmented repression of the CcpA regulon.

The connection between Mn and sugar catabolism is not unprecedented, as previous studies have implicated Mn as important for the activity of carbon catabolism enzymes in other bacteria (Kehres and Maguire, 2003). Additionally, recent studies in *S. pneumoniae* and *S. mutans* have shown that fluctuations in metal homeostasis influence the regulation of carbohydrate metabolism (Burcham et al., 2020; Kajfasz et al., 2020). One possible explanation for the Mn-dependent, glucose-independent CcpA repression observed in our study is the accumulation of the glycolytic intermediate, fructose-1,6-bisphosphate (FBP) during Mn depletion (Figure 7). In Firmicutes, phosphorylation of histidine phosphocarrier protein (HPr) to HPr(Ser-P) occurs when FBP and ATP levels are high (Gorke and Stulke, 2008). HPr(Ser-P) then binds to CcpA, which in turn induces the binding of the repressor to *cre* sites on the DNA. Additionally, FBP enhances the binding interaction of HPr(Ser-P) and CcpA, increasing repression. In our concurrent metabolomics study, we found that Δ*ssaACB* cells accumulated FBP at T_50_ after Mn depletion (Puccio et al., 2020), which may explain the strong evidence for CcpA repression. As expected if CcpA were responsible for the changes in expression, we found that 48 out of the 169 DEGs found by Bai et al. (2019) when comparing a Δ*ccpA* mutant to the SK36 WT were changed in the opposite direction as our T_50_ sample. However, 15 significant DEGs were in the same direction, and the remainder were unchanged in our study. Additionally, most of the DEGs we observed in our study did not overlap with those of Bai et al. (2019). This comparison indicates that CcpA-dependent repression could be responsible for some of the changes in expression after Mn depletion, but it does not explain all of the observed results.

To determine potential causes for the accumulation, we examined the enzymes required for synthesizing and catabolizing FBP (Figure 7A). Both enzymes responsible for metabolizing FBP, fructose-1,6-bisphosphatase (Fbp; SSA_1056) and fructose-bisphosphate aldolase (Fba; SSA_1992), may require a Mn cofactor according to BRENDA (RRID:SCR_002997)^1^ (Jeske et al., 2019). Thus, it is possible that Mn depletion led to reduced activity of both Fba and Fbp and this resulted in accumulation of FBP (Puccio et al., 2020), which in turn induced CcpA repression after Mn depletion (Figure 7B). Enzymatic activity assays will be required to determine the true cofactor for these enzymes in *S. sanguinis*, but accumulation of FBP is strong evidence that the activity of at least one enzyme in this pathway is affected by Mn depletion.

### Reduced (p)ppGpp Hydrolase Activity May Contribute to the Post-Mn Depletion Phenotype

The relationship between (p)ppGpp and carbon source utilization has been previously established; *S. mutans* strains lacking RelA or all Rel proteins showed delayed growth rates when transitioned from media containing glucose to lactose (Zeng et al., 2018). When we examined the genome for *cre* sites, *relQ* was predicted to have a 5’ *cre* site and was downregulated at T_50_ (Supplementary Table S2), indicating that it could be under CcpA control. Little is known about the transcriptional regulation of *relA* in streptococci (Nascimento et al., 2008; Irving and Corrigan, 2018), although regulation of activity has been established in other species (Gratani et al., 2018). In *S. mutans*, expression of *relP* is activated by a two-component system, RelRS, which is thought to sense oxidative stressors (Seaton et al., 2011). It was hypothesized by Kim et al. (2012) that (p)ppGpp production by RelP in *S. mutans* may be an attempt by the cell to slow growth to minimize damage from oxygen radicals produced during metabolism. While it was observed in this study that H_2_O_2_ levels decreased in response to Mn depletion, it is possible that other ROS were present due to a decrease in SodA activity. Thus, increased expression and activity of RelP, in addition to lack of hydrolase activity by RelA, could be at least partly responsible for the reduced growth rate.

In analyzing previous transcriptome studies using Δ*relA* or *rel*^0^ mutants in the related species *S. mutans, S. pneumoniae*, and *Enterococcus faecalis* (Nascimento et al., 2008; Kazmierczak et al., 2009; Colomer-Winter et al., 2019), we noted many expression patterns similar to our study. While these studies utilized different species and growth conditions and thus are not a direct comparison, it is remarkable that reduction in (p)ppGpp levels would lead to similar changes in gene expression as Mn depletion. Of interest to us, several PTS genes were downregulated in all three previous studies. Similar to the results we observed in this study, the *S. pneumoniae* Δ*relA* mutant showed decreased expression of *spxB* and *sodA* (Kazmierczak et al., 2009). These comparisons indicate either that dysregulation of (p)ppGpp levels leads to changes in expression of these genes in response to stress or that decreased activity of the Rel hydrolase domain due to Mn depletion is not responsible for the observed changes in expression of these genes in *S. sanguinis*.

Based on these results, we hypothesize that reduced activity of the RelA hydrolase domain may contribute to the observed reduction in growth rate in the fermentor studies but is not entirely responsible. Specifically, our inability to eliminate the cell’s only known (p)ppGpp hydrolase, combined with our finding that the Δ*relA* strains, which also have no hydrolase, exhibited worse growth than *rel*^0^ mutants having no synthetase and therefore no (p)ppGpp, suggests that (p)ppGpp accumulation is highly detrimental to growth. A definitive test of this hypothesis will require measurement of (p)ppGpp levels in fermentor-grown cells. We are currently assessing various approaches for feasibility. In addition, the significant decrease in growth of the Δ*ssaACB* Δ*relA* strain as compared to the Δ*ssaACB* parent shows that there is an additive effect, indicating that the impact of the loss of RelA is not entirely Mn-dependent.

## Conclusion

The effect of Mn depletion on a multitude of diverse systems indicates that the impact of Mn is not relegated to only a few enzymes. Depletion of Mn does not induce a traditional stress response, instead inducing what appears to be dysregulation of many different genes that leads to rapid reduction in the growth rate, despite plentiful nutrients and other metals. While decreased function of the known Mn-cofactored enzymes, such as NrdF, SodA, and the hydrolase domain of RelA, likely contributed to the decreased growth rate we observed upon Mn depletion, it is probably a combination of multiple systems leading to the observed phenotype. Additionally, many of the affected systems appear to be regulated by CCR through CcpA-dependent repression in a glucose-independent manner. Future research will focus on determining the respective contribution of each putative Mn-dependent enzyme as well as whether there is a direct relationship between Mn and CCR.

## Materials and Methods

### Bacterial Strains and Growth Conditions

The *S. sanguinis* strain SK36 is a human oral isolate from Mogens Killian, Aarhus University, Denmark. All mutant strains were generated in the SK36 background (Table 2) using the primers listed in Supplementary Table S3. The Δ*ssaACB* strains were generated previously (Baker et al., 2019) with all three genes replaced with either a kanamycin (Kan) resistance gene, *aphA-3*, or tetracycline (Tet) resistance gene, *tetM*, using gene splicing by overlap extension (SOEing) PCR (Ho et al., 1989). The Δ*ssaACB*::*aphA-3* mutation is non-polar, as confirmed by complementation (Murgas et al., 2020). With the exception of Figure 5, all Δ*ssaACB* experiments were completed with the Kan^*R*^ strain, JFP169. The Δ*relA*, Δ*relP*, and Δ*relQ* mutants were re-created for this study by amplifying the *aphA-3* gene and flanking DNA from the corresponding, previously created mutants (Xu et al., 2011) using primers from the same study. All PCR products were purified using a Qiagen MinElute PCR kit prior to transformation. Transformations employing antibiotic selection were performed using the protocol described previously (Paik et al., 2005). Briefly, an overnight culture of the parent strain was grown in BD Bacto^TM^ Todd Hewitt broth with horse serum (Invitrogen), then diluted 200-fold and incubated at 37°C. Optical density (OD_600_) of tube cultures was determined using a Thermo Scientific BioMate 3S UV-VIS spectrophotometer. Knockout construct DNA (100 ng) and *S. sanguinis* competence stimulating peptide (70 ng) were added to the culture (OD_600_ ∼0.07) and incubated at 37°C for 1.5 h prior to selective plating on BHI (BD) agar plates with antibiotics. Kan (Sigma-Aldrich) was added to a final concentration of 500 μg mL^–1^. All plates were incubated for 24 h at 37°C under anaerobic conditions, where atmospheric composition was adjusted using a programmable Anoxomat Mark II jar-filling system (AIG, Inc.) and a palladium catalyst was included in the jars. All mutants were confirmed to have the expected composition by sequence analysis of the DNA flanking the insertion sites.

**TABLE 2:** Strains used in this study.

Strain	Description	Source or reference
SK36	Human oral plaque isolate	M. Killian, Aarhaus University; Xu et al., 2007
JFP132	Δ*sodA*::*aphA-3*	Crump et al., 2014
JFP169^†^	Δ*ssaACB*::*aphA-3*	Baker et al., 2019
JFP173^‡^	Δ*ssaACB*::*tetM*	Baker et al., 2019
SSX_0250	Δ*relA*::*aphA-3*	Xu et al., 2011
JFP259	Δ*relA*::*aphA-3*	This study
JFP260	Δ*ssaACB*::*tetM* Δ*relA::aphA-3*	This study
SSX_1210	Δ*relQ*::*aphA-3*	Xu et al., 2011
JFP279	Δ*relQ*::*aphA-3*	This study
JFP281	Δ*ssaACB*::*tetM* Δ*relQ::aphA-3*	This study
SSX_1795	Δ*relP*::*aphA-3*	Xu et al., 2011
JFP275	Δ*relP*::*aphA-3*	This study
JFP277	Δ*ssaACB*::*tetM* Δ*relP::aphA-3*	This study
JFP276	Δ*relA*::*aphA-3*Δ*relP*	This study
JFP278	Δ*ssaACB*::*tetM* Δ*relA*::*aphA-3*Δ*relP*	This study
JFP280	Δ*relA*::*aphA-3*Δ*relP*Δ*relQ* (*rel*^0^)	This study
JFP282	Δ*ssaACB*::*tetM* Δ*relA*::*aphA-3*Δ*relP*Δ*relQ* (*rel*^0^)	This study
SK36 IFDC	pheS* ermAM	Cheng et al., 2018

**^†^Designated ΔssaACB throughout the manuscript. ^‡^Only used for ΔssaACB strains in Figure 5.**

To generate the *rel*^0^ strain, Δ*relP* and Δ*relQ* mutations were made using a markerless mutation system described previously (Xie et al., 2011; Cheng et al., 2018). Briefly, the IFDC2 cassette was amplified from the *S. sanguinis* IFDC2 strain and combined with flanking region from *relP* using gene SOEing. The two parent Δ*relA* strains (WT and Δ*ssaACB*::*tetM* backgrounds) were then transformed as described above, plating on BHI agar plates containing 10 μg mL^–1^ erythromycin (Erm; Fisher Scientific). A gene SOEing product merging the two flanking regions of *relP* was then generated. This SOEing product was then used to transform the Erm^*R*^ colonies from the first transformation. Immediately prior to plating on agar plates containing 20 mM 4-chloro-phenylalanine (Sigma-Aldrich), the cells were washed twice with phosphate-buffered saline (PBS) to remove excess media. Resulting colonies were then screened for Erm sensitivity and sequenced to confirm removal of the desired gene and IFDC2 cassette. The process was then repeated for *relQ*, converting the two Δ*relA* Δ*relP* parent strains into *rel*^0^ strains.

Overnight BHI cultures (pre-cultures) were inoculated from single-use aliquots of cryopreserved cells by 1,000-fold dilution. Antibiotics were included in mutant pre-cultures at the aforementioned concentrations or 5 μg mL^−1^ for Tet (Sigma Aldrich). Cultures were then incubated for approximately 18 h at 6% O_2_ (Anoxomat jar set to 6% O_2_, 7% H_2_, 7% CO_2_, and 80% N_2_) at 37°C. To determine CFUs, samples were sonicated for 90 s using an ultrasonic homogenizer (Biologics, Inc) to disrupt chains prior to dilution in PBS and plated using an Eddy Jet 2 spiral plater (Neutec Group, Inc.). For static growth studies, tubes containing 100% pooled rabbit serum (Gibco) were pre-incubated at 37°C in an atmosphere of 6% O_2_. Each tube was then inoculated with a 10^–6^-fold dilution of the overnight pre-culture, as described above. The inoculated tubes were returned to incubate at the same oxygen concentration. Cultures were removed after 24 h, sonicated, and diluted in PBS prior to plating on BHI agar. Plates were incubated for 24 h in 0% O_2_ prior to colony enumeration.

### Fermentor Growth Conditions and Sample Collection

A BIOSTAT B bioreactor (Sartorius Stedim) with a 1.5-L capacity UniVessel glass vessel^®^ was used for growth of 800-mL cultures at 37°C, as described in Puccio and Kitten (2020). Briefly, cultures were stirred at 250 rpm and pH was maintained by the automated addition of 2 N KOH (Fisher Chemical). A 40-mL overnight pre-culture of *S. sanguinis* was grown as described above and centrifuged for 10 min at 3,740 × *g* in an Allegra X-142 centrifuge at 4°C (Beckman-Coulter). The supernatant was discarded and the cells were resuspended in BHI prior to inoculation. The air flow was increased stepwise, based on the OD of the fermentor culture. At the peak OD, air flow was increased to 0.50 liters per min, input flow of fresh BHI was set to 17% (∼700 mL h^–1^) and output flow of waste was set to 34%. Cells were allowed to acclimate to the new conditions for 1 h. The T_–__20_ sample was aseptically removed for total RNA isolation or metal analysis (described below). EDTA (Invitrogen) was introduced to the carboy 16 mins later (T_–__4_) to achieve a final concentration of 100 μM. EDTA was then introduced directly to the vessel 4 min later (T_0_), corresponding to the time at which EDTA from the carboy would reach the vessel, to achieve a final concentration of 100 μM. Samples were taken for each post-EDTA time point (T_10_, T_25_, T_50_). In some experiments, MnSO_4_ or FeSO_4_ (Puratronic^TM^; Alfa Aesar) was added to the carboy (T_66_) and vessel (T_70_) at a final concentration of 100 μM and samples were taken for metal analysis at T_80_.

### Metal Analysis

Additional 40-mL cell culture samples were collected from WT and Δ*ssaACB* cells at the same fermentor growth time points as described above. The cells were immediately centrifuged at 3,740 × *g* for 10 min at 4°C. The supernatant was decanted and the cell pellet was washed twice with cold cPBS (PBS treated with Chelex-100 resin (Bio-Rad) for 2 h, then filter sterilized and supplemented with EDTA to 1 mM). The pellet was then divided for subsequent acid digestion or protein concentration determination. Trace metal grade (TMG) nitric acid (15%) (Fisher Chemical) was added to one portion of the pellet. The pellet was digested using an Anton Paar microwave digestion system using a modified Organic B protocol: 120°C for 10 min, 180°C for 20 min, with the maximum temperature set to 180°C. The digested samples were then diluted 3-fold with Chelex-treated dH_2_O. Metal concentrations were determined using an Agilent 5110 inductively coupled plasma-optical emission spectrometer (ICP-OES). Concentrations were determined by comparison with a standard curve created with a 10 μg mL^–1^ multi-element standard (CMS-5; Inorganic Ventures) diluted in 5% TMG nitric acid. Pb (Inorganic Ventures) was used as an internal standard (10 μg mL^–1^). The other portion of the pellet was resuspended in PBS and mechanically lysed using a FastPrep-24 instrument with Lysing Matrix B tubes (MP Biomedicals) as described previously (Rhodes et al., 2014). Insoluble material was removed by centrifugation. Protein concentrations were determined using a bicinchoninic acid Protein Assay Kit (Pierce) as recommended by the manufacturer, with bovine serum albumin as the standard. Absorbance was measured in a black, flat-bottom 96-well plate (Greiner) using a microplate reader (BioTek).

### Total RNA Isolation

For each RNA sample, 2 mL of fermentor culture was added to 4 mL RNAprotect Bacteria Reagent (Qiagen) and immediately vortexed for 10 s. The sample was then incubated at room temperature for 5–90 min. The samples were then centrifuged for 10 min at 3,740 × *g* at 4°C. The supernatant was discarded and the samples stored at −80°C. RNA isolation and on-column DNase treatment were completed using the RNeasy Mini Kit and RNase-Free DNase Kit, respectively (Qiagen). RNA was eluted in 50 μL RNase-Free water (Qiagen). A second DNase treatment was then performed on the samples (Invitrogen). Total RNA was quantified and purity was assessed using a Nanodrop 2000 Spectrophotometer (Thermo Fisher Scientific).

### RNA-Seq Library Preparation and Sequencing

Total RNA quantity and integrity were determined using an Agilent Bioanalyzer RNA Pico assay. All samples passed quality control assessment with RNA Integrity Numbers (RIN) above 8. Two sequential rounds of ribosomal reduction were then performed on all samples using Illumina’s Ribo-Zero rRNA Removal Kit. The resulting depleted RNA was assessed using an Agilent Bioanalyzer RNA Pico assay to confirm efficient rRNA removal. Stranded RNA-seq library construction was then performed on the rRNA-depleted RNA using the Ultra II Directional RNA Library Prep Kit for Illumina (New England Biolabs), following manufacturer’s specifications for library construction and multiplexing. Final Illumina libraries were assessed for quality using an Agilent Bioanalyzer DNA High Sensitivity Assay and qPCR quantification was performed using NEBNext Library Quant kit for Illumina (New England Biolabs). Individual libraries were pooled equimolarly, and the final pool was sequenced on an Illumina MiSeq, with 2 × 150-bp paired-end reads. Demultiplexing was performed on the Illumina MiSeq’s on-board computer and resulting demultiplexed files uploaded to Illumina BaseSpace for data delivery. The University of Virginia Department of Biology Genomics Core Facility (Charlottesville, Virginia) completed all RNA-seq library preparation and sequencing.

### RNA-Seq Analysis Pipeline

Using Geneious 11.1 (RRID:SCR_010519)^2^, sequence reads were paired and then trimmed using the BBDuk Trimmer prior to mapping to a modified SK36 genome, in which the *ssaACB* operon was replaced with the *aphA-3* sequence. The locus tags are from the Genbank annotation (Benson et al., 2013) available at the time; the annotations were updated shortly before publication and the new locus tags are included in Supplementary Table S1 for reference. PATRIC annotations (RRID:SCR_004154)^3^ (Wattam et al., 2017) are also included for reference. Reads for each post-EDTA sample were compared to the corresponding pre-EDTA (T_–__20_) sample using DESeq2 (Love et al., 2014) (RRID: SCR_015687) in Geneious to determine log_2_ fold changes and adjusted *P*-values. Principal component analysis was completed using R (version 3.6.1) and RStudio (version 1.2.5033-1) with Bioconductor (Bioconductor, RRID:SCR_006442) package pcaExplorer version 2.13.0 (Marini and Binder, 2019). Volcano plots were generated using R and RStudio with Bioconductor package EnhancedVolcano (Blighe et al., 2018). All DEGs were input into the DAVID database (RRID:SCR_001881)^4^ (Dennis et al., 2003). The KEGG_pathway option was chosen for functional annotation clustering. The *P*-value shows the significance of pathway enrichment. Figure 2C was generated using an R script^5^.

### Hydrogen Peroxide Quantification

Culture supernatants without RNAprotect were collected at each time point and stored at −20°C. Hydrogen peroxide concentration was measured using a Fluorometric Hydrogen Peroxide Assay Kit (Sigma). Standards were prepared from 3% hydrogen peroxide provided with the kit as recommended by the manufacturer. Fluorescence was measured in a black-walled, flat-bottom 96-well plate (Greiner) using a microplate reader (BioTek).

### Data Analysis and Presentation

All statistical tests, excluding RNA-seq DESeq2 calculations, were performed in GraphPad InStat^6^. Significance was determined by analysis of variance (ANOVA) as indicated in the figure legends. For ANOVA, a Tukey-Kramer test for multiple comparisons was used when *P* ≤ 0.05. DESeq2 calculations were completed in Geneious 11.1 or in the pcaExplorer R package, as indicated above. Confidence intervals (95%) of replicate samples were determined by the pcaExplorer R package. *P* ≤ 0.05 were considered significant. All graphs and the heat map were constructed using GraphPad Prism^6^; Figure 7 was made using Biorender.com.

## Data Availability Statement

The data discussed in this publication have been deposited in NCBI’s Gene Expression Omnibus (Edgar et al., 2002) and are accessible through GEO Series accession number GSE150593 (https://www.ncbi.nlm.nih.gov/geo/query/acc.cgi? acc=GSE150593).

## Author Contributions

TP and TK designed the experiments and wrote all drafts of the manuscript. TP and KK performed the experiments. TP, KK, and BZ generated the figures. TP, TK, KK, BZ, and PX analyzed the data. All authors reviewed and approved the final version of the manuscript.

## Conflict of Interest

The authors declare that the research was conducted in the absence of any commercial or financial relationships that could be construed as a potential conflict of interest.

## Footnotes

1 brenda-enzymes.org

2 https://www.geneious.com

3 https://patricbrc.org/

4 https://david.ncifcrf.gov/summary.jsp

5 https://github.com/DrBinZhu/DAVID_FIG

6 graphpad.com

## Supplementary Material

Supplementary Table 1 RNA-seq Analysis.

Supplementary Table 2 Analysis of putative *cre* sites and CcpA regulon.

Supplementary Table 3 Primers used in this study.

Supplementary Data Sheet 1 Figures, Methods, and References.
