# Supplementary material for: Manganese Depletion Leads to Multisystem Changes in the Transcriptome of the Opportunistic Pathogen Streptococcus sanguinis
Source: Front Microbiol. 2020 Nov 5;11:592615. doi: 10.3389/fmicb.2020.592615 (PMC7674665; doi:10.3389/fmicb.2020.592615)
Supplement: Supplementary Data Sheet 1 — Figures, Methods, and References. [file Data_Sheet_1.pdf]

Supplementary Material

1 Supplementary Figures

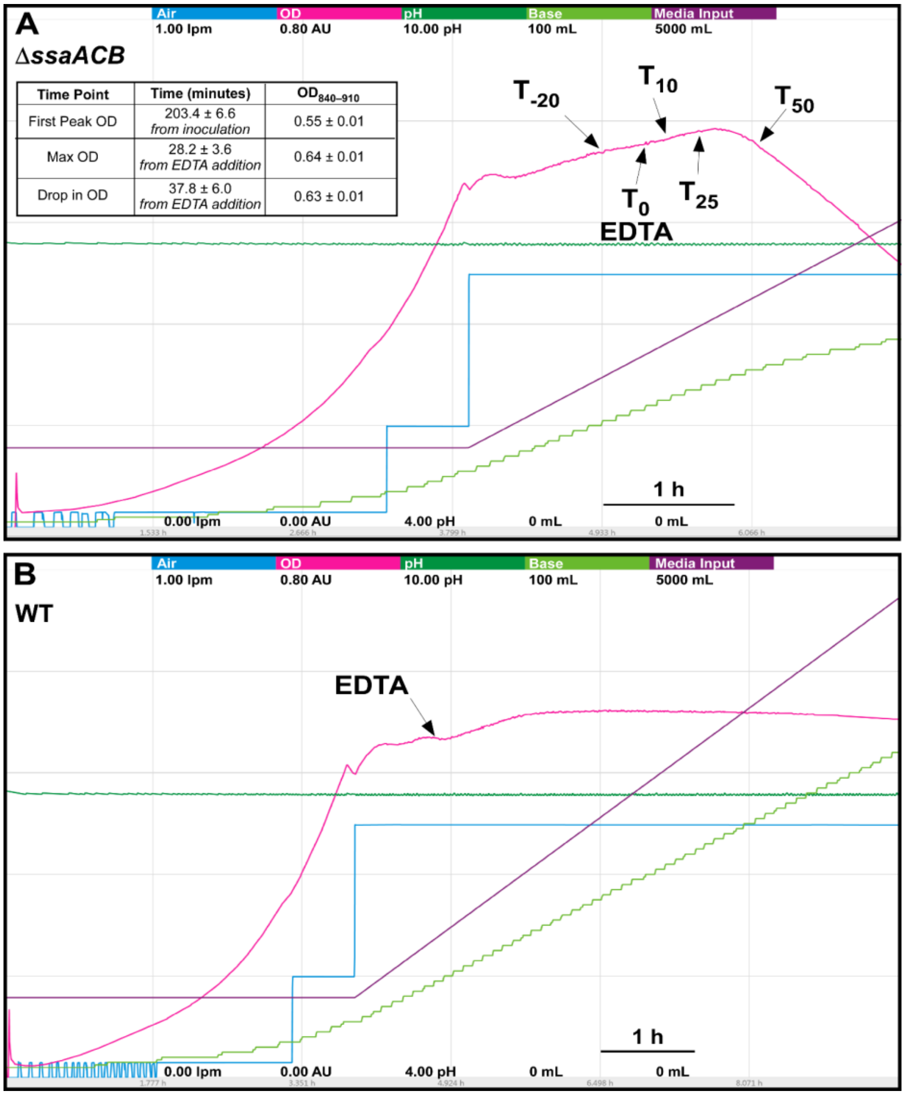

Figure S1 | Aerobic fermentor growth of Δ*ssaACB* and WT strains. Representative charts for fermentor growth of *S. sanguinis* (**A**) Δ*ssaACB* and (**B**) WT cells. Each color represents a different parameter: cyan - air flow (liters per min; lpm), pink - optical density (840-910 nm; absorbance units; AU), dark green - pH, light green - base input (KOH), purple - media input (mL; total volume). Each color represents a different parameter as labeled at the top of the figure. The scale for each parameter is indicated by the values under each respective parameter label (minimum at the bottom, maximum at the top). The time scale is indicated by the bar in the bottom right portion of each chart. Cells were grown under aerobic conditions with EDTA added 80 mins (T_0) after the media input and output pumps were turned on and the air flow was set to 0.5 lpm. Each sample time point is labeled.

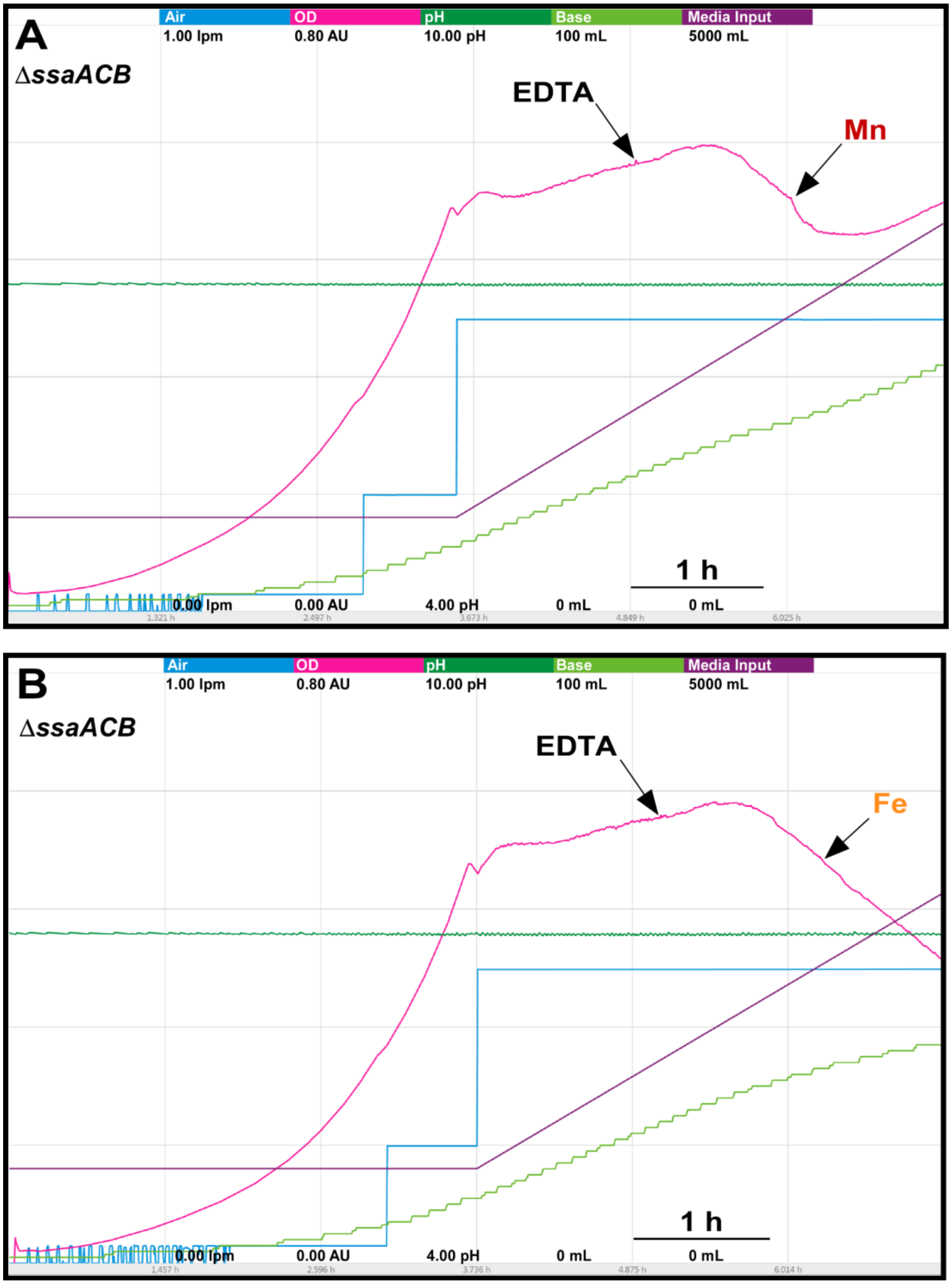

Figure S2 | Addition of metals to fermentor-grown Δ*ssaACB* cells post-EDTA. Fermentor growth of Δ*ssaACB* with the addition of 100 µM EDTA at T_0 as described previously, with 100 µM of either Mn^{2+} (**A**) or Fe^{2+} **(B)** added at T_{70}. Colors and labels are as in Fig. S1. The time scale is indicated by the bar in the bottom right portion of the each chart. Each chart is representative of at least three replicates.

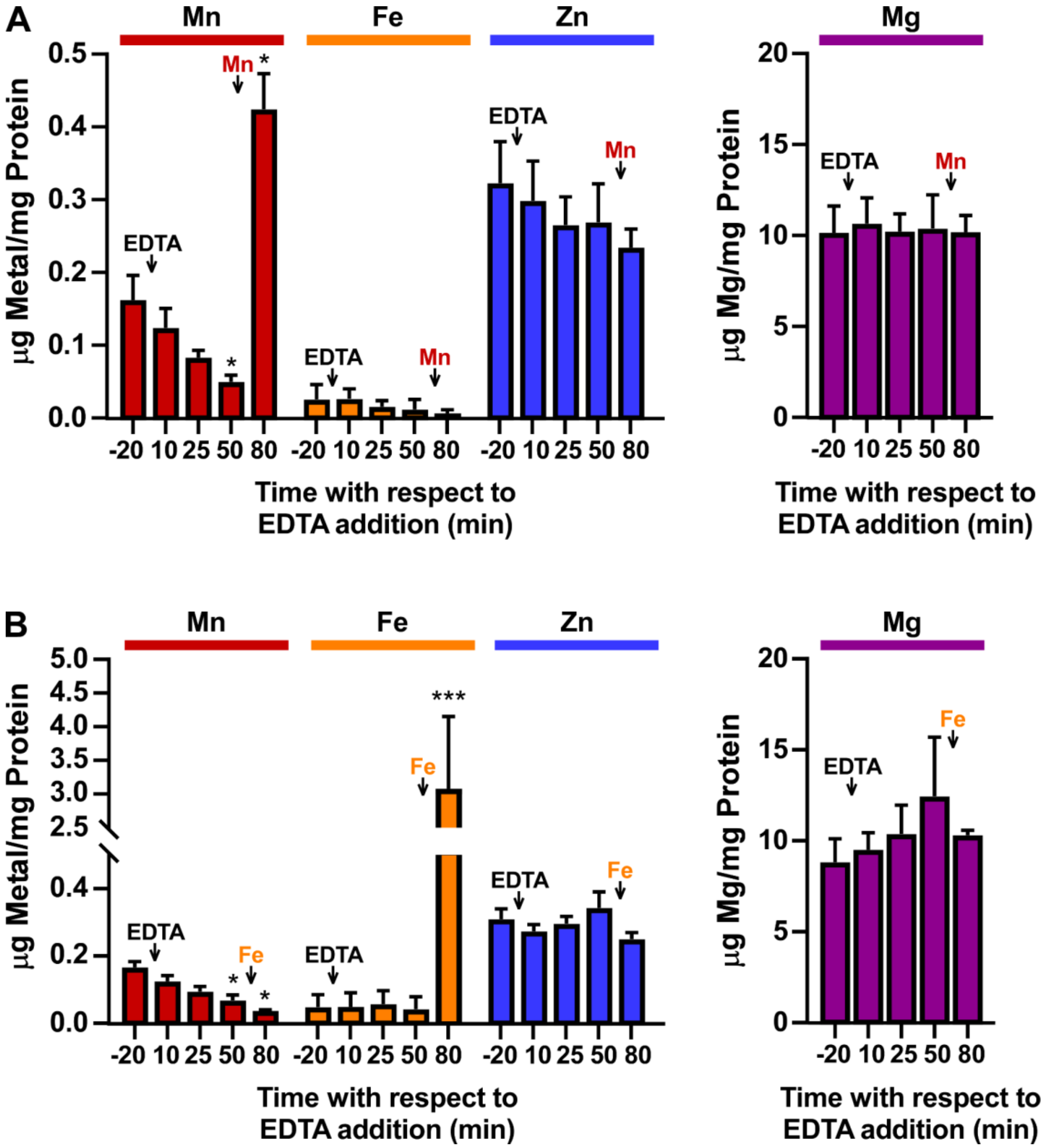

Figure S3 | Metal content of fermentor-grown Δ*ssaACB* cells post-EDTA and metal supplementation. Samples of Δ*ssaACB* cells were collected from the fermentor at each time point and analyzed for cellular metal content using ICP-OES. The T_{80} time point is 10 mins after the addition of 100 µM of either Mn^{2+} (**A**) or Fe^{2+} **(B)** added at T_{70} as depicted in Figure S2. Means and standard deviations of three replicates are shown. Significance was determined for each metal by repeated measures ANOVA or one-way ANOVA if matching was not effective. A Tukey-Kramer multiple comparisons test was used for comparison to T_{-20} for each metal; **P* ≤ 0.05, ****P* ≤ 0.0001.

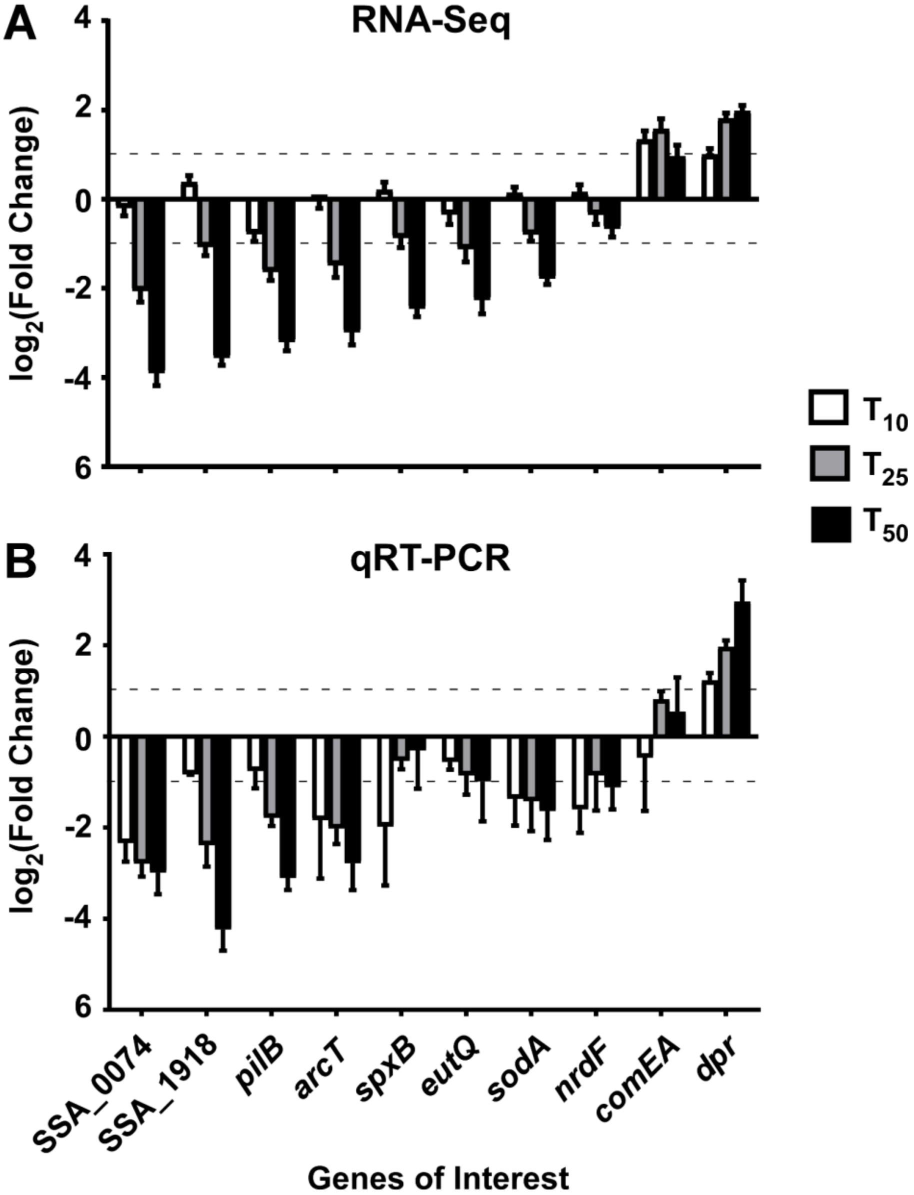

Figure S4 | Validation of RNA-Seq trends using qRT-PCR**.** (**A**) The average $\log_2$ fold change values of select genes of interest from the DESeq2 RNA-Seq analysis, comparing each post-EDTA time point to T_{-20}. The average is from four biological replicates. (**B**) Log_2 fold change values of the same genes as determined by qRT-PCR of two additional Δ*ssaACB* fermentor run samples. Mean and standard error for each time point are depicted. Horizontal dashed lines indicate $\log_2$ fold changes in expression of ± 1.

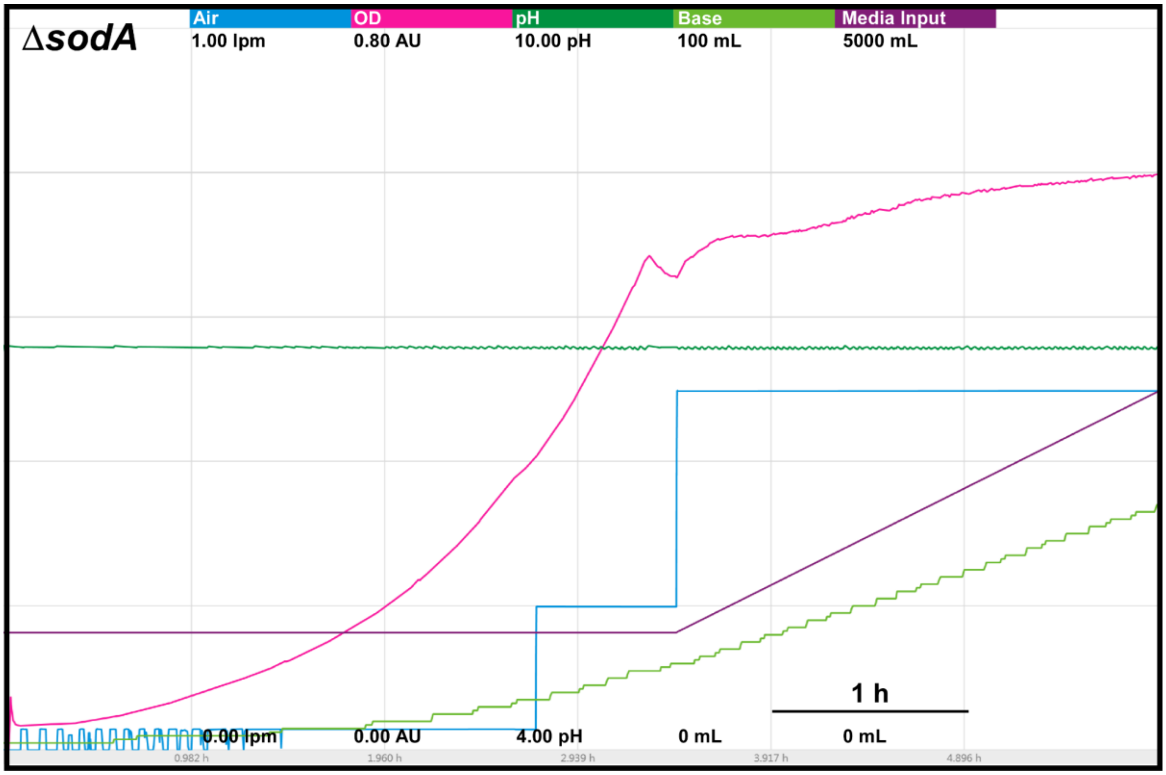

Figure S5 | Aerobic fermentor growth of the Δ*sodA* mutant. The Δ*sodA* mutant grown under aerobic fermentor conditions as described previously, without EDTA. Each color represents a different parameter, labeled at the top of the figure. The scale for each parameter is indicated by the values under each respective parameter (minimum at the bottom, maximum at the top). The time scale is indicated by the bar in the bottom right portion of the chart. Representative chart from three replicates.

Locus Tag	Annotation	TPM	Log₂ Ratio		
		T_{-20}	T_{10}/T_{-20}	T_{25}/T_{-20}	T_{50}/T_{-20}
SSA_0363	*dagA*, sodium:alanine symporter family protein	120	0.23	**1.26**	**1.22**
SSA_0364	*dctA*, serine/threonine transporter	102	0.30	**1.16**	**1.14**
SSA_0369	NADP oxidoreductase	767	0.52	**1.02**	**1.68**
SSA_0370	GNAT family N-acetyltransferase	1070	0.48	**1.00**	**1.61**
SSA_0371	*gdhA*, NADP-specific glutamate dehydrogenase	1079	0.29	**0.84**	**1.41**
SSA_0385	*opuAb*, ABC transporter permease/substrate binding protein	275	-0.03	**-1.09**	**-1.74**
SSA_0386	*opuAa* , glycine betaine/L-proline ABC transporter ATP-binding protein	287	-0.26	**-1.32**	**-2.00**
SSA_0572	2,3-butanediol dehydrogenase	147	0.10	-0.19	**-1.75**
SSA_0635	*trpC* , indole-3-glycerol phosphate synthase	18	0.26	-0.02	**-1.03**
SSA_0637	*trpB* , tryptophan synthase subunit beta	55	**1.22**	**1.25**	**1.38**
SSA_0638	*trpA* , tryptophan synthase subunit alpha	83	**1.30**	**1.28**	**1.07**
SSA_0703	citrate synthase	4	-0.10	0.56	**1.54**
SSA_0704	NADP-dependent isocitrate dehydrogenase	7	-0.51	0.44	**1.03**
SSA_0737	*sagP* , arginine deiminase	292	**1.17**	0.09	**-1.92**
SSA_0738	*arc*, ornithine carbamoyltransferase	666	**0.47**	**-0.93**	**-2.40**
SSA_0739	*arcC* , carbamate kinase	58	0.24	**-1.45**	**-3.15**
SSA_0741	*arcT* , acetylornithine deacetylase/succinyl-diaminopimelate desuccinylase	76	-0.01	**-1.49**	**-2.99**
SSA_0917	5-methyltetrahydropteroyltriglutamate-homocysteine methyltransferase	5	0.17	0.33	**1.34**
SSA_0921	*adhB* , alcohol dehydrogenase catalytic domain-containing protein	128	-0.04	**0.75**	**1.23**
SSA_1341	*carB* , carbamoyl-phosphate synthase large subunit	23	-0.03	**0.65**	**1.31**
SSA_1342	*carA* , glutamine-hydrolyzing carbamoyl-phosphate synthase small subunit	20	0.27	**0.77**	**1.26**
SSA_1360	amino acid ABC transporter ATP-binding protein	724	**0.50**	**0.81**	**1.16**
SSA_1401	PLP-dependent aminotransferase family protein	17	-0.09	0.24	**1.05**
SSA_1439	*hisK* , hypothetical protein	73	**0.62**	**0.78**	**1.41**
SSA_1440	*hisE* , phosphoribosyl-ATP diphosphatase	75	0.16	0.47	**1.11**
SSA_1569	amino acid ABC transporter permease	112	**0.77**	**0.91**	**1.01**
SSA_1615	alanine dehydrogenase	368	**0.88**	-0.35	**-3.45**
SSA_1621	amino acid permease	80	0.28	**-1.19**	**-3.17**
SSA_1713	*serA* , D-3-phosphoglycerate dehydrogenase	51	0.36	**1.37**	**2.22**
SSA_1715	*serC* , 3-phosphoserine/phosphohydroxythreonine transaminase	40	0.05	**1.07**	**2.03**
SSA_1949	peptide ABC transporter substrate-binding protein	182	**0.40**	**-0.95**	**-2.27**
SSA_1950	peptide ABC transporter ATP-binding protein	95	-0.16	**-0.68**	**-1.38**
SSA_1967	*ilvA* , threonine ammonia-lyase	58	0.21	**0.81**	**1.32**
SSA_1968	*ilvC* , ketol-acid reductoisomerase	210	0.31	**0.88**	**1.39**
SSA_1969	*ilvH* , acetolactate synthase small subunit	108	-0.09	0.57	**1.09**
SSA_1970	*ilvB* , acetolactate synthase large subunit	114	-0.34	**0.49**	**1.01**
SSA_2039	amino acid permease	23	0.01	**-0.97**	**-1.18**
SSA_2141	*argH* , argininosuccinate lyase	14	0.06	0.39	**1.24**

Log₂ Ratio
2.5
2.0
1.5
1.0
0.5
0.0
-0.5
-1.0
-1.5
-2.0
-2.5
-3.0
-3.5
-4.0
-4.5

Figure S6 | Impact of Mn depletion on amino acid transport and synthesis genes. Expression of amino acid transport and synthesis genes in the Δ*ssaACB* mutant are depicted with their average TPM at T_{-20} and $\log_2$ fold change values for each post-EDTA time point. Only genes with $|\log_2$ fold change values$|$ ≥ 1 are depicted in this chart. For expression of all amino acid transport and synthesis genes, see Table S1. TPM values greater than 1000 are full saturation (green). Positive $\log_2$ fold change values (red) are upregulated in post-EDTA samples as compared to T_{-20}, while negative values (blue) are downregulated. Values in bold are significant by adjusted *P*-value (≤ 0.05).

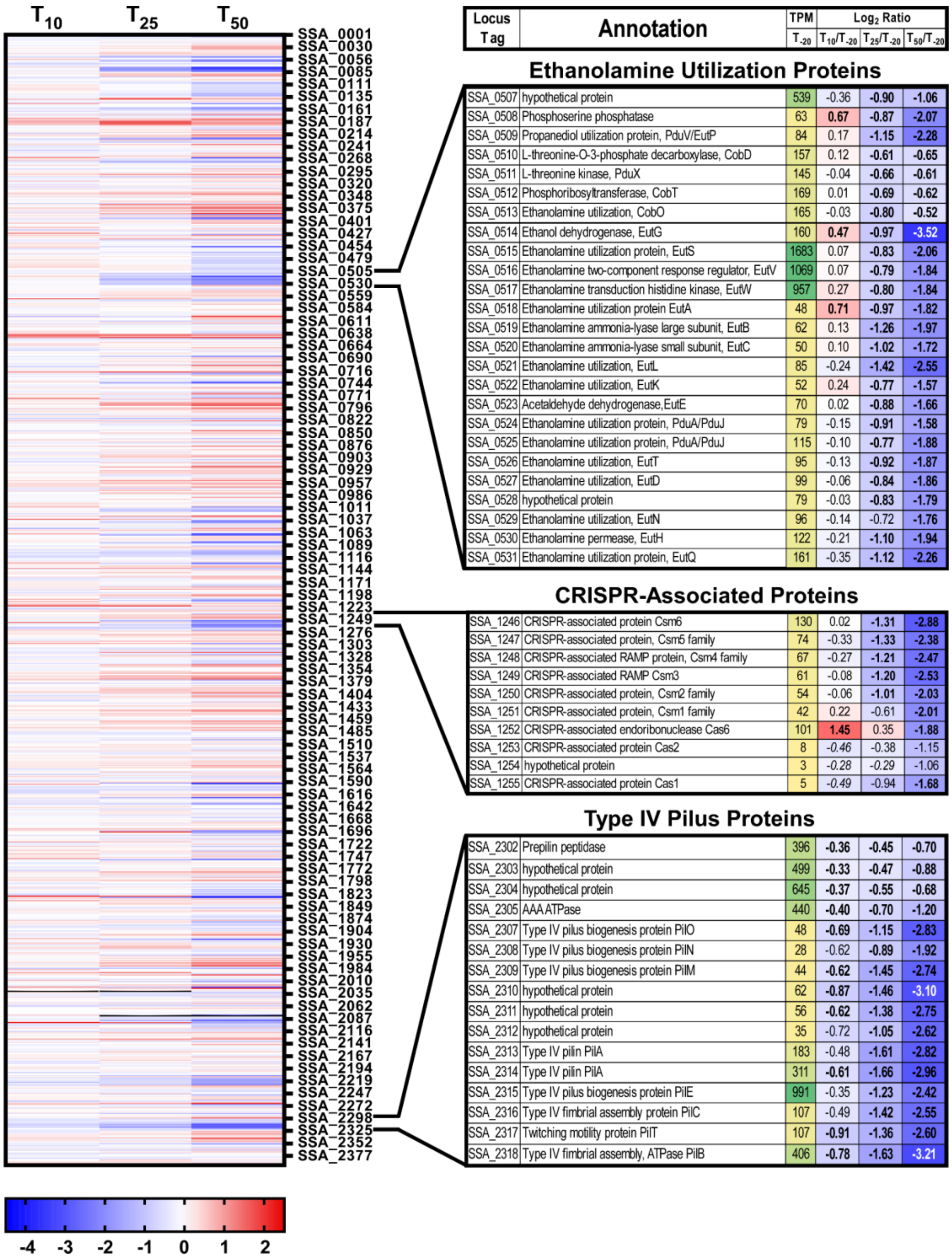

Locus Tag	Annotation	TPM	Log₂ Ratio		
		T_{-20}	T_{10}/T_{-20}	T_{25}/T_{-20}	T_{50}/T_{-20}
	**Ethanolamine Utilization Proteins**				
SSA_0507	hypothetical protein	539	-0.36	**-0.90**	**-1.06**
SSA_0508	Phosphoserine phosphatase	63	**0.67**	**-0.87**	**-2.07**
SSA_0509	Propanediol utilization protein, PduV/EutP	84	0.17	**-1.15**	**-2.28**
SSA_0510	L-threonine-O-3-phosphate decarboxylase, CobD	157	0.12	**-0.61**	**-0.65**
SSA_0511	L-threonine kinase, PduX	145	-0.04	**-0.66**	**-0.61**
SSA_0512	Phosphoribosyltransferase, CobT	169	0.01	**-0.69**	**-0.62**
SSA_0513	Ethanolamine utilization, CobO	165	-0.03	**-0.80**	**-0.52**
SSA_0514	Ethanol dehydrogenase, EutG	160	**0.47**	**-0.97**	**-3.52**
SSA_0515	Ethanolamine utilization protein, EutS	1683	0.07	**-0.83**	**-2.06**
SSA_0516	Ethanolamine two-component response regulator, EutV	1069	0.07	**-0.79**	**-1.84**
SSA_0517	Ethanolamine transduction histidine kinase, EutW	957	0.27	**-0.80**	**-1.84**
SSA_0518	Ethanolamine utilization protein EutA	48	**0.71**	**-0.97**	**-1.82**
SSA_0519	Ethanolamine ammonia-lyase large subunit, EutB	62	0.13	**-1.26**	**-1.97**
SSA_0520	Ethanolamine ammonia-lyase small subunit, EutC	50	0.10	**-1.02**	**-1.72**
SSA_0521	Ethanolamine utilization, EutL	85	-0.24	**-1.42**	**-2.55**
SSA_0522	Ethanolamine utilization, EutK	52	0.24	**-0.77**	**-1.57**
SSA_0523	Acetaldehyde dehydrogenase,EutE	70	0.02	**-0.88**	**-1.66**
SSA_0524	Ethanolamine utilization protein, PduA/PduJ	79	-0.15	**-0.91**	**-1.58**
SSA_0525	Ethanolamine utilization protein, PduA/PduJ	115	-0.10	**-0.77**	**-1.88**
SSA_0526	Ethanolamine utilization, EutT	95	-0.13	**-0.92**	**-1.87**
SSA_0527	Ethanolamine utilization, EutD	99	-0.06	**-0.84**	**-1.86**
SSA_0528	hypothetical protein	79	-0.03	**-0.83**	**-1.79**
SSA_0529	Ethanolamine utilization, EutN	96	-0.14	-0.72	**-1.76**
SSA_0530	Ethanolamine permease, EutH	122	-0.21	**-1.10**	**-1.94**
SSA_0531	Ethanolamine utilization protein, EutQ	161	-0.35	**-1.12**	**-2.26**
	**CRISPR-Associated Proteins**				
SSA_1246	CRISPR-associated protein Csm6	130	0.02	**-1.31**	**-2.88**
SSA_1247	CRISPR-associated protein, Csm5 family	74	-0.33	**-1.33**	**-2.38**
SSA_1248	CRISPR-associated RAMP protein, Csm4 family	67	-0.27	**-1.21**	**-2.47**
SSA_1249	CRISPR-associated RAMP Csm3	61	-0.08	**-1.20**	**-2.53**
SSA_1250	CRISPR-associated protein, Csm2 family	54	-0.06	**-1.01**	**-2.03**
SSA_1251	CRISPR-associated protein, Csm1 family	42	0.22	-0.61	**-2.01**
SSA_1252	CRISPR-associated endoribonuclease Cas6	101	**1.45**	0.35	**-1.88**
SSA_1253	CRISPR-associated protein Cas2	8	*-0.46*	-0.38	-1.15
SSA_1254	hypothetical protein	3	*-0.28*	*-0.29*	-1.06
SSA_1255	CRISPR-associated protein Cas1	5	*-0.49*	-0.94	**-1.68**
	**Type IV Pilus Proteins**				
SSA_2302	Prepilin peptidase	396	**-0.36**	**-0.45**	**-0.70**
SSA_2303	hypothetical protein	499	**-0.33**	**-0.47**	**-0.88**
SSA_2304	hypothetical protein	645	**-0.37**	**-0.55**	**-0.68**
SSA_2305	AAA ATPase	440	**-0.40**	**-0.70**	**-1.20**
SSA_2307	Type IV pilus biogenesis protein PilO	48	**-0.69**	**-1.15**	**-2.83**
SSA_2308	Type IV pilus biogenesis protein PilN	28	-0.62	**-0.89**	**-1.92**
SSA_2309	Type IV pilus biogenesis protein PilM	44	**-0.62**	**-1.45**	**-2.74**
SSA_2310	hypothetical protein	62	**-0.87**	**-1.46**	-3.10
SSA_2311	hypothetical protein	56	**-0.62**	**-1.38**	**-2.75**
SSA_2312	hypothetical protein	35	-0.72	**-1.05**	**-2.62**
SSA_2313	Type IV pilin PilA	183	-0.48	**-1.61**	**-2.82**
SSA_2314	Type IV pilin PilA	311	**-0.61**	**-1.66**	**-2.96**
SSA_2315	Type IV pilus biogenesis protein PilE	991	-0.35	**-1.23**	**-2.42**
SSA_2316	Type IV fimbrial assembly protein PilC	107	-0.49	**-1.42**	**-2.55**
SSA_2317	Twitching motility protein PilT	107	**-0.91**	**-1.36**	**-2.60**
SSA_2318	Type IV fimbrial assembly, ATPase PilB	406	**-0.78**	**-1.63**	-3.21

Figure S7 | Transcriptomic heatmap of Δ*ssaACB* aerobic fermentor grown cells**.** Heatmap displaying the $\log_2$ fold change values of each gene at the time indicated as compared to T_{-20}. Positive $\log_2$ fold change values (red) are upregulated in later samples as compared to T_{-20}, while negative values (blue) are downregulated. Select genes are depicted with their average TPM at T_{-20} and $\log_2$ fold change values for each post-EDTA time point. TPM values greater than 1000 are full saturation (green). Log_2 fold change values follow the same color scale as depicted in the heatmap. Values in bold are significant by adjusted *P*-value (≤ 0.05).

2 Supplementary Methods

Fermentor set up

BHI was prepared in a polypropylene carboy to a final volume of 5 L. Antifoam (Sigma) was added to 25 ppm and the carboy was autoclaved for 2 h at 128°C. Aeration was achieved by the controlled addition of compressed N_2 and air delivered through a ring sparger, augmented by stirring at 250 rpm; vessel temperature was controlled via an external heating blanket. The DO, OD, and temperature were also measured by indwelling probes. Constant volume in the vessel was maintained by the placement of the opening of a harvest tube at the 800 mL level. Detailed protocol is described in Puccio and Kitten (2020).

Quantitative reverse transcriptase polymerase chain reaction

RNA was collected as described in the main text. cDNA libraries were created using SensiFAST cDNA Synthesis Kit (Bioline). Control reactions without reverse transcriptase were conducted to confirm the absence of contaminating DNA in all samples. qRT-PCR was performed using SYBR Green Supermix (Applied Biosystems) on an Applied Biosystems 7500 Fast Real Time PCR System using the primers listed in Table S3. Relative gene expression was analyzed using the $2^{-\Delta\Delta CT}$ method (Livak and Schmittgen, 2001) with *gapA* serving as the internal control (Rodriguez et al., 2011).

Heatmap generation

The heatmap was generated in GraphPad Prism[1] using the $\log_2$ fold change values of the RNA-Seq data calculated using DESeq2 in Geneious[2], as described in the main text.

Putative *cre* site identification

Putative *cre* sites identified in the SK36 genome by RegPrecise[3] (RRID:SCR_002149) (Novichkov et al., 2013) and listed within the "propagated regulon" collection[4] were collected. Further analyses were performed by obtaining non-overlapping sequences ≤ 250 bp in length upstream of all SK36 genes using RSAT[5] (RRID:SCR_008560) (van Helden et al., 2000; Nguyen et al., 2018), then searching for putative *Streptococcus suis* pseudopalindromic *cre* and *cre*2 motifs (Willenborg et al., 2014) in these sequences using FIMO from MEME Suite[6] (RRID:SCR_001783) (Grant et al., 2011). Motifs used for each search, as well as scores given for the RegPrecise and FIMO outputs, are listed in Table S2. Due to the variable length of the cre_{var} sites (Yang et al., 2017), seqinR v. 3.6-1 (Charif and Lobry, 2007) was used for this search. The FIMO cutoff was set to P-value $\leq 10^{-4}$. Putative sites located within 10 bp of the start site of the corresponding gene were removed from the list.

---

[1] https://www.graphpad.com/

[2] https://www.geneious.com/

[3] https://enigma.lbl.gov/regprecise/

[4] http://regprecise.sbpdiscovery.org:8080/WebRegPrecise/regulon.jsp?regulon_id=35148

[5] http://rsat.sb-roscoff.fr/

[6] http://meme-suite.org/doc/fimo.html

3 Supplementary References

Charif, D., and Lobry, J.R. (2007). "SeqinR 1.0-2: A contributed package to the R project for statistical computing devoted to biological sequences retrieval and analysis," in *Structural Approaches to Sequence Evolution: Molecules, Networks, Populations,* eds. U. Bastolla, M. Porto, H.E. Roman & M. Vendruscolo. (Berlin, Heidelberg: Springer Berlin Heidelberg), 207-232.

Grant, C.E., Bailey, T.L., and Noble, W.S. (2011). FIMO: scanning for occurrences of a given motif. *Bioinformatics* 27(7)**,** 1017-1018. doi: 10.1093/bioinformatics/btr064.

Livak, K.J., and Schmittgen, T.D. (2001). Analysis of relative gene expression data using real-time quantitative PCR and the $2^{(-\Delta\Delta CT)}$ method. *Methods* 25(4)**,** 402-408. doi: 10.1006/meth.2001.1262.

Nguyen, N.T.T., Contreras-Moreira, B., Castro-Mondragon, J.A., Santana-Garcia, W., Ossio, R., Robles-Espinoza, C.D., et al. (2018). RSAT 2018: regulatory sequence analysis tools 20th anniversary. *Nucleic Acids Res* 46(W1)**,** W209-w214. doi: 10.1093/nar/gky317.

Novichkov, P.S., Kazakov, A.E., Ravcheev, D.A., Leyn, S.A., Kovaleva, G.Y., Sutormin, R.A., et al. (2013). RegPrecise 3.0 - A resource for genome-scale exploration of transcriptional regulation in bacteria. *BMC Genomics* 14**,** 745. doi: 10.1186/1471-2164-14-745.

Puccio, T., and Kitten, T. (2020). Fermentor growth of *Streptococcus sanguinis*. *protocols.io*. doi: dx.doi.org/10.17504/protocols.io.bkayksfw.

Rodriguez, A.M., Callahan, J.E., Fawcett, P., Ge, X., Xu, P., and Kitten, T. (2011). Physiological and molecular characterization of genetic competence in *Streptococcus sanguinis*. *Mol Oral Microbiol* 26(2)**,** 99-116. doi: 10.1111/j.2041-1014.2011.00606.x.

van Helden, J., André, B., and Collado-Vides, J. (2000). A web site for the computational analysis of yeast regulatory sequences. *Yeast* 16(2)**,** 177-187. doi: 10.1002/(sici)1097-0061(20000130)16:2.

Willenborg, J., de Greeff, A., Jarek, M., Valentin-Weigand, P., and Goethe, R. (2014). The CcpA regulon of *Streptococcus suis* reveals novel insights into the regulation of the streptococcal central carbon metabolism by binding of CcpA to two distinct binding motifs. *Mol Microbiol* 92(1)**,** 61-83. doi: 10.1111/mmi.12537.

Yang, Y., Zhang, L., Huang, H., Yang, C., Yang, S., Gu, Y., et al. (2017). A flexible binding site architecture provides new insights into CcpA global regulation in gram-positive bacteria. *mBio* 8(1). doi: 10.1128/mBio.02004-16.
